# Predicting antibacterial activity from snake venom proteomes

**DOI:** 10.1371/journal.pone.0226807

**Published:** 2020-01-24

**Authors:** Justin L. Rheubert, Michael F. Meyer, Raeshelle M. Strobel, Megan A. Pasternak, Robert A. Charvat

**Affiliations:** 1 Department of Biology, University of Findlay, Findlay, Ohio, United States of America; 2 School of the Environment, Washington State University, Pullman, Washington, United States of America; Instituto Butantan, BRAZIL

## Abstract

The continued evolution of antibiotic resistance has increased the urgency for new antibiotic development, leading to exploration of non-traditional sources. In particular, snake venom has garnered attention for its potent antibacterial properties. Numerous studies describing snake venom proteomic composition as well as antibiotic efficacy have created an opportunity to synthesize relationships between venom proteomes and their antibacterial properties. Using literature reported values from peer-reviewed studies, our study generated models to predict efficacy given venom protein family composition, snake taxonomic family, bacterial Gram stain, bacterial morphology, and bacterial respiration strategy. We then applied our predictive models to untested snake species with known venom proteomic compositions. Overall, our results provide potential protein families that serve as accurate predictors of efficacy as well as promising organisms in terms of antibacterial properties of venom. The results from this study suggest potential future research trajectories for antibacterial properties in snake venom by offering hypotheses for a variety of taxa.

## Introduction

The intensifying threat of antibiotic resistance to human health has led to a burgeoning of studies concerning new antibiotics. Before 1990 (including the golden years of antibiotic discovery, 1940s-1960s), 13% of antibiotic literature was concerned with resistance (based on analysis of publications in PubMed with the keywords 'antibiotic' and 'resistance’). Following 1990, literature pertaining to antibiotic resistance expanded more than two-fold (31%), highlighting the growing problem of resistance and necessitating the hunt for novel compounds [1,2]. One source of potential therapeutic value, venom, gained the attention of many researchers due to its high level of cellular toxicity [3]. Research on snake venoms in particular has uncovered numerous antimicrobial agents which include but are not limited to: L-amino acid oxidases (LAAOs), phospholipase A_2_s (PLA_2_s), hyaluronidases (HYAs), and snake venom metalloproteinases (svMPs) [4,5].

Crude venoms and their constituents have previously been determined to be effective against bacteria. For example, venom extracted from *Ophiophagus hannah* was shown to hinder *Pseudomonas aeruginosa*, *Salmonella enteritidis*, *Escherichia coli*, and *Staphylococcus aureus* growth [6,7], including resistant strains such as Methicillin-resistant *S*. *aureus* (MRSA). Numerous studies focused on the antibacterial properties of snake venom with over 58% of those studies reporting effective results [8]. Furthermore, individual components of snake venom have been isolated and tested for their antibacterial properties with the two most commonly studied protein families being PLA_2_ and LAAO [4].

Although venoms are generally associated with antibacterial effectiveness, venoms from different snake species display markedly different effectiveness towards various bacteria. For example, crude venom from *Crotalus atrox* is effective against *E*. *coli*, whereas crude venom from *Crotalus durissus* is not [9]. This difference may be attributed to variance in venom composition such as the high concentrations of svMPs in *C*. *atrox* [10] and low concentrations in *C*. *durissus* [11]. Although venom composition may be similar between taxa, certain protein families may be limited to a select group of snakes, such as 3-finger toxins (3FTXs) mainly limited to the Elapidae. Furthermore, the overall composition of the venom may differ in terms of the proportion of its constituents, such as svMPs in *C*. *atrox* (49.7%; [10]) and *C*. *durissus* (4.8%; [11]).

Although effective components within venom have been isolated and tested for antibacterial efficacy [3,12,13], syntheses concerning the overall relationship between venom composition and antibacterial effectiveness are lacking. Anecdotal evidence indicates differences in snake venom effectiveness exist at the family level, with Viperidae crude venom being more effective against certain bacterial classes (i.e., Gram-negative) than the Elapidae [8]. Furthermore, antibacterial testing of snake venom currently lacks quantitative direction to guide future studies concerning which venoms may have the highest probability of efficacy. The purpose of this study is two-fold: 1) to generate predictive models using snake species with known venom proteomic composition and antibacterial effectiveness, and 2) to apply well performing models in instances where snake venom protein composition is known but antibacterial effectiveness is unknown.

## Methods

Our methodology can be described in four main steps. First, we created a database of snake venom protein compositions and associated antibacterial effectiveness from previously published works. Second, we generated statistical models using only snake species that had both described venom proteomes as well as data regarding the venom’s antibacterial efficacy. Third, models were cross-validated using model fit and accuracy as performance criteria. Finally, we used well performing, fitted models to predict antibacterial effectiveness for species with known venom protein composition and unknown antibacterial effectiveness.

### 2.1. Description of the data

An exhaustive literature search concerning venom proteomic analyses was completed by using keywords including “snake”, “venom”, “proteome”, “composition”, and “protein” (as well as their derivatives) to search online journal repositories including EBSCOHost, Google Scholar, and PubMed. Recognizing that differences in database search algorithms may have excluded some studies from our purview, bibliographies within each relevant and related article were also examined. Articles that appeared in these bibliographies but absent from our original search were also included for analysis. This process continued until no unique articles were identified. Only studies that included original, complete proteomic percentages of venom components were used, culminating 172 subjects regardless of age, sex, and/or population (see supplemental references). Snake species, snake family, and percent of each protein family were recorded. Protein families of less than 0.1% of the total proteome were not recorded as they were reported as <0.1% in the original works and exact values were not reported. Individual protein families included 3FTXs, 5’nucleotidases (5’NUCs), bradykinin potentiating peptides (BPPs), cysteine rich secretory proteins (CRISPs), C-type lectins (CTLs), disintegrins (DISs), HYAs, LAAOs, PLA_2_s, snake venom growth factors (svGFs), svMPs, snake venom peptides (svPEPs), snake venom serine proteinases (svSPs), vespryn/ohanins (VESP/OHAs), waglerins (WAGs), and waprin/kunitz type inhibitors (WAP/KUNs). Individual protein families appearing less than five times in the completed database were omitted to reduce variation from less frequently reported protein families. Furthermore, small organic molecules that may be present in the venom were omitted because of the absence of their reports in proteomic studies and the limited amount of data presently available on their presence and role in venoms.

To incorporate antibacterial effectiveness data, previous works concerning antibacterial properties of snake venom (gathered using the same methods as described above except using keywords “snake”, “venom”, “antibacterial”, “antimicrobial”, and “bacterial efficacy”) were compiled for the bacteria tested, where venoms were either effective or not effective. Published works were assessed, detailing 924 antibacterial efficacy tests of crude venom regardless of concentration utilized (see supplemental references). Additional bacterial metadata included Gram stain, respiration strategy (i.e., aerobic, anaerobic, and facultative anaerobic), and morphology. Due to differences in data presentation and methodologies, efficacy was reported based on the original authors’ interpretation of the data. After comparing snake species present in both venom composition and bacterial efficacy data sets, 28 snake species (n = 505 across snake families) had both described venom protein composition data as well as efficacy data. These 28 species were then used for model selection and validation. When aggregating venom compositions and antibacterial efficacies, intraspecific proteomic compositions were averaged, though we are cognizant that intraspecific variation does occur. To assess that interspecific variation in venom proteomes was greater than intraspecific variation, we performed a PERMANOVA with venom composition as a response to snake species. Because models were ultimately built for the Elapidae and Viperidae species, we performed PERMANOVAs solely on Elapidae (p = 0.002; S1 Fig) and Viperidae (p = 0.001; S2 Fig). The remaining data, which included unknown antibacterial activity were then used for predicted efficacies following model selection.

### 2.2. Model generation and cross-validation

Using the 28 snake species for which we had complete venom proteomes and antibacterial effectiveness data, we applied an exhaustive model selection technique with logistic regression models that predicted antibacterial effectiveness as a function of arcsine-square root transformed proportional protein composition [14]. While venom protein data were neither univariate nor multivariate normally distributed (assessed by Kolmogorov-Smirnov for univariate and Mardia and Henze-Zinkler for multivariate), logistic regressions have shown robustness to departures from normality [15]

Certain protein families were systematically eliminated prior to model selection by comparing interspecific variance of protein family proportion. A protein family with a low interspecific variance (i.e., < 0.01 or 1% of the proteome) was *a priori* deemed as a poor predictor of antibacterial effectiveness as many instances in which a protein family only constituted less than 1% of the entire proteome were not reported in original works. Similarly, we also removed protein families with low variance to mean ratios (i.e., < 5% of the interspecific mean) as certain proteins, although varying greater than 1% of the proteome, may marginally change proteomic compositions relative to their average proportions. For example, while serine proteinases had an interspecific variance of 0.017 for Viperidae, they also had a mean interspecific proportion of 0.36. Consequently, serine proteinase variation is likely minute in comparison to the protein families’ typical proportion, and its inclusion in model selection may only add additional noise to the model selection process. Finally, protein families that strongly covaried (i.e., Pearson’s Correlation p < 0.01) were removed from model selection, such that all protein family predictors were independent [16].

Following exhaustive model generation, models were first assessed using Akaike Information Criteria (AICc) values [17], where the best performing models were defined as those within 2 AICc points [18]. Second, pseudo-R^2^ [19] was then calculated for each model in order to assess goodness of fit. As a third measure of model performance, we calculated the area under the curve (AUC) of the receiver operator characteristics (ROC) curve [20]. The ROC curve graphs the variance in the rate of an event occurring with the rate of a falsely predicted event. An AUC of 0.5 indicates a false prediction rate increases 1:1 with the rate of a correct prediction. AUCs greater than 0.5 imply a model performing better than random (sensu [21]). To test significance of AUC values, effectiveness responses were permuted with fixed venom compositions, and then AUC was recalculated. This process was repeated 1,000 times in order to create a randomized distribution of AUC. The original AUC value was then compared to the distribution of permuted AUCs. The proportion of AUCs greater than or equal to the original AUC by chance is considered the p-value of a particular model. Among all possible models generated, the model with the lowest AICc, highest pseudo-R^2^, and highest AUC was deemed the best performing model. Model coefficients are summarized in S1 Table.

Models fitted from known antibacterial efficacies were assessed based on a confusion matrix scheme, which details True Positives, True Negatives, False Positives, and False Negatives. True results are defined as results that are congruent with model predictions. False results are defined as those when model predictions and reality are not congruent. Model accuracy is defined as the ratio of the sum of True Positives and True Negatives to the Total Outcomes [22].

Given the above criteria for model selection and validation, we ranked relative model performance as “good” or “poor”. A “good” model had a high accuracy (i.e., > 70%) and a significant p-value (p < 0.05). A “poor” model had neither high accuracy nor a significant p-value. A model accuracy threshold of 70% was selected to account for variation in model performance that may result from small sample sizes as opposed to actually being inaccurate [23,24]. While a threshold of 70% is less than thresholds for studies employing large datasets [25], our accuracy threshold enables models with small sample sizes to not be excluded from providing potentially insightful predictions for antibiotic efficacy of certain species.

For all “good” models, we performed a holdout cross-validation technique to assure that models were not overfit to the data used to develop the model. Holdout cross-validation is a technique in which randomly selected data are removed from model generation, also called the “training set” [22]. Data not included in the “training set” were included in a “test set”. Models were regenerated using the “training set”, and then model accuracy was recalculated using the “test set”. We created training sets with 80% of the total data and test sets with the remaining 20%. This process was iterated 1,000 times so as to create a distribution of accuracies. The original accuracy was then compared to the distribution of accuracies. If the original accuracy was not in the top 5% (i.e., p > 0.05) of accuracies reported from the holdout cross-validation, we considered the model to not be overfit.

Models were subset based on snake family (i.e., Viperidae and Elapidae), bacterial Gram stain, bacterial respiration strategy, and bacterial morphology because differences in venom composition based on relatedness of snake families were expected *a priori*. Models with data subset by solely Gram stain, morphology, respiration strategy, and snake family were attempted, but failed to produce robust accuracies; therefore, combinatory groups based off these classifications were created.

### 2.3. Prediction of antibacterial effectiveness for unknown species

For all “good” models that were not deemed as overfit to our data, we predicted antibacterial effectiveness for snake species with unknown antibacterial activity. Snake species’ venom with greater than 50% predicted probability of effectiveness were considered as having antibacterial potential. The majority of species were from Elapidae and Viperidae families. We also predicted antibacterial effectiveness for three Colubridae species, for which the data were only applied to well performing Elapidae and Viperidae models. Predictions were not made using models that were deemed “poor” as these models may lead to erroneous conclusions. However, readers are encouraged to add in newly acquired data and test these models using the supplied R-scripts.

### 2.4. Supplemental methods and data information

All models were generated and validated within the R environment for statistical computing [26]. An accompanying technical document as well as raw data, aggregated data, and R scripts are available to assist in future implementation of these methods [27]. The raw data file contains original citations for each data point collected. The technical document is an R markdown script, written in a vignette style, which provides additional details about the R code used in this study. As future studies provide additional information about venom composition and antibacterial effectiveness, the script can be iterated by interested users to update the model.

## Results

### 3.1. Elapidae venom

#### 3.1.1. Gram-positive facultative anaerobic bacillus bacteria

These analyses utilized PLA_2_, svMP, and WAP/KUN values (n = 30; 13 unique species). Following exhaustive model selection, our results suggest the best predictive model was an intercept-only model based off AICc (S3A Fig). Model 2 also offered a marginally better pseudo-R^2^ as well as AUC (S3A Fig). Successive models were less competitive to the null model with respect to AICc, but were not as rigorous as Model 2 with respect to pseudo-R^2^ and AUC. For these reasons, we identified Model 2 as the best predictive model (S2A Fig). Because Model 2’s AUC and pseudo-R^2^ marginally contrasted with the null model, Model 2’s accuracy to discriminate efficacy was only 66.67%. Post-hoc AUC permutations of efficacies indicate a non-significant model (S3C Fig; p = 0.279). This is most likely because the model produces high rates of false negatives, where nine of the ten true positives were classified as negative (S2B Fig). Together, these results suggest that the model does not effectively discriminate antibiotic efficacy, and we consider this model as performing poorly.

#### 3.1.2. Gram-positive facultative anaerobic coccus bacteria

**Fig 1:**
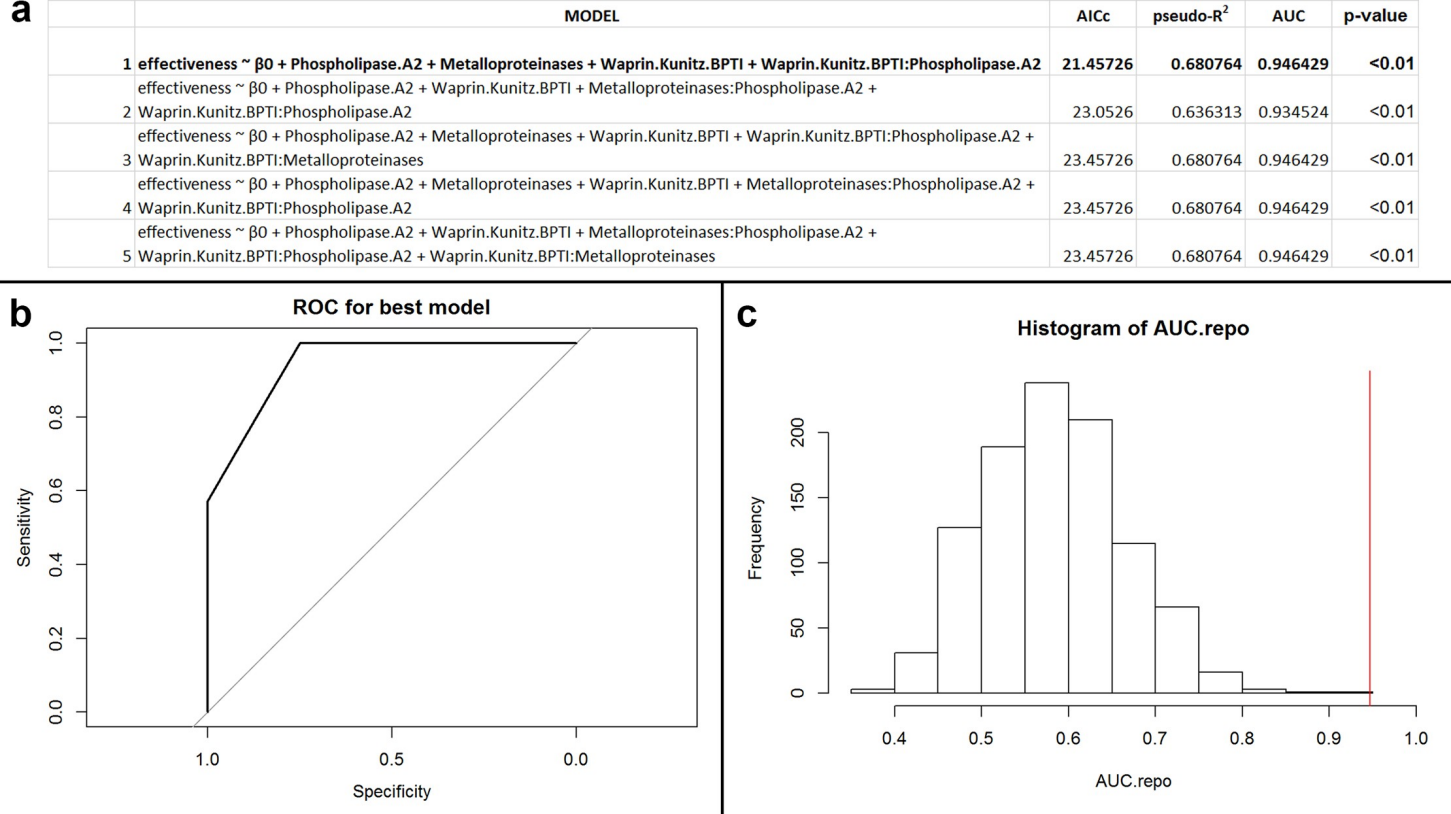
(a) Table of performances for the best five models that predict antibacterial efficacy using PLA_2_s, svMPs, and WAP/KUNs for Elapidae snakes against Gram-positive, facultative anaerobic, coccus bacteria. The variable β_0_ indicates the intercept value for the model. Models defined as ‘effectiveness ~ β_0_’ are intercept models. Model 1, the best performing model, is in bold. (b) Receiver operator characteristic (ROC) curve for best performing model (Model 1). The area under the curve (AUC) of the ROC informs goodness of fit, where a value greater than 0.5 indicates the model performs better than random. In this instance, the AUC of the best performing model is 0.95. (c) Histogram of recalculated AUC values from permuted data. The red vertical line indicates the position of the observed AUC value from the non-permuted data. The number of AUC values greater than the original value is the exact p-value for the best performing model (Model 1). In this case, the p-value of the best performing model is <0.01.

These analyses (n = 26; 12 unique species) implemented PLA_2_, svMP, and WAP/KUN. Our results demonstrate that the best predictive model was Model 1 (Fig 1A). Because of this model’s high accuracy of 88.5%, the only misclassifications consisted of false positives (three of 26 samples; Fig 1B). Together, these results indicate that Model 1 effectively discriminates antibiotic efficacy, and we consider this model to be performing well. Post-hoc holdout analysis additionally suggested that the model may not be overfit to our data subset (Fig 1C; p = 0.15).

#### 3.1.3. Gram-negative aerobic bacillus bacteria

This set (n = 32; 12 unique species) incorporated PLA_2_, svMP, and WAP/KUN. Our model selection methods demonstrated that the null model was the best predictive model by AICc (S4A Fig), whereas Model 2 was more robust with respect to pseudo-R^2^ and AUC (S4A Fig). Because two of our three criteria support Model 2 as the better model, we performed successive analyses using Model 2. Our post-hoc permutations demonstrated Model 2 had an accuracy of 56.7% and produced an especially high false negative rate, where 12 of 14 effective venoms were predicted as ineffective (S4B Fig). These combined results suggest that Model 2 does not effectively discriminate antibiotic efficacies and is, therefore, a poorly performing model (S4C Fig; p = 0.44).

#### 3.1.4. Gram-negative facultative anaerobic bacillus bacteria

**Fig 2:**
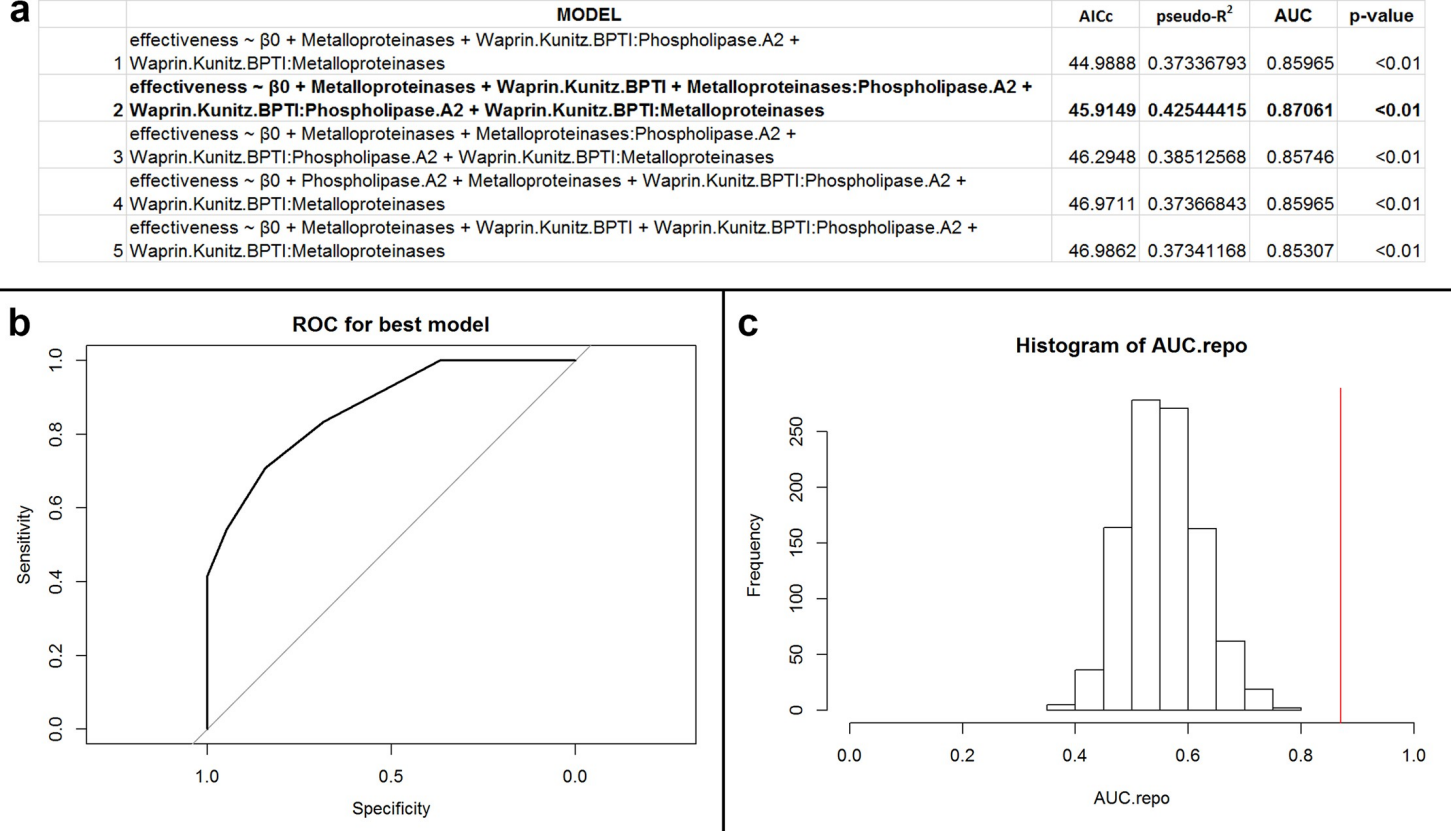
(a) Table of performances for the best five models that predict antibacterial efficacy using PLA_2_s, svMPs, and WAP/KUNs for Elapidae snakes against Gram-negative, facultative anaerobic, bacillus bacteria. The variable β_0_ indicates the intercept value for the model. Models defined as ‘effectiveness ~ β_0_’ are intercept models. Model 2, the best performing model, is in bold. (b) Receiver operator characteristic (ROC) curve for best performing model (Model 2). The area under the curve (AUC) of the ROC informs goodness of fit, where a value greater than 0.5 indicates the model performs better than random. In this instance, the AUC of the best performing model is 0.87. (c) Histogram of recalculated AUC values from permuted data. The red vertical line indicates the position of the observed AUC value from the non-permuted data. The number of AUC values greater than the original value is the exact p-value for the best performing model (Model 2). In this case, the p-value of the best performing model is <0.01.

Protein families for this subset (n = 43; 13 unique species) included PLA_2_, svMP, and WAP/KUN (S1 Table). Model 1 was most competitive based off AICc (Fig 2A). However, Model 2 had more rigorous pseudo-R^2^ and AUC values (Fig 2A). Model 2 was overall highly significant (Fig 2C; p < 0.01) with an accuracy of 76.7% (Fig 2B). Together, our results indicate that Model 2 discriminates antibiotic efficacy, and we consider this model as performing well. Post-hoc accuracy analysis suggested the data may not be overfit to the data subset (p = 0.32).

### 3.2. Viperidae venom

#### 3.2.1. Gram-positive facultative anaerobic bacillus bacteria

**Fig 3:**
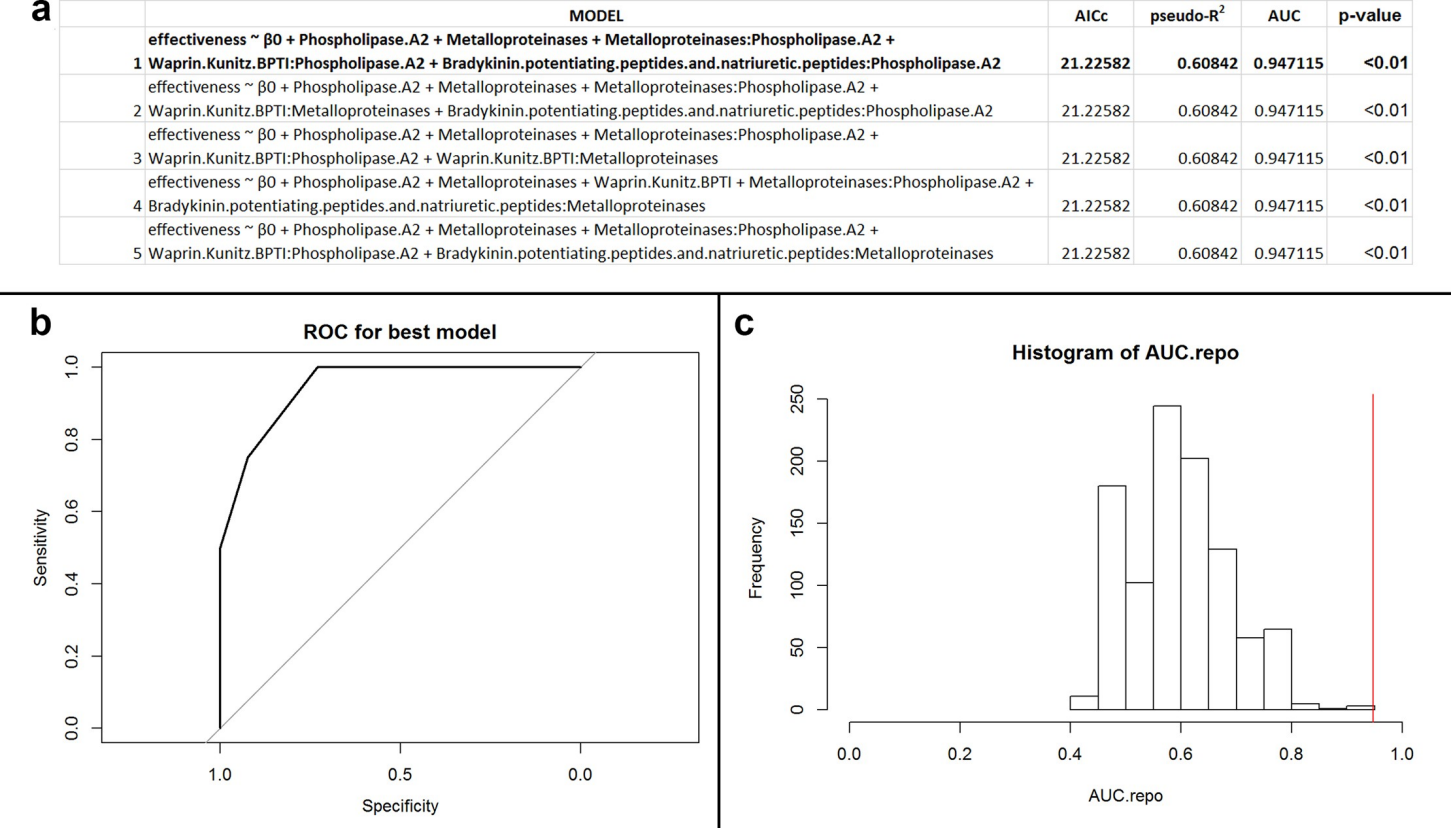
(a) Table of performances for the best five models that predict antibacterial efficacy using PLA_2_s, svMPs, BPPs, and WAP/KUNs for Viperidae snakes against Gram-positive, facultative anaerobic, bacillus bacteria. The variable β_0_ indicates the intercept value for the model. Models defined as ‘effectiveness ~ β_0_’ are intercept models. Model 1, the best performing model, is in bold. (b) Receiver operator characteristic (ROC) curve for best performing model (Model 1). The area under the curve (AUC) of the ROC informs goodness of fit, where a value greater than 0.5 indicates the model performs better than random. In this instance, the AUC of the best performing model is 0.95. (c) Histogram of recalculated AUC values from permuted data. The red vertical line indicates the position of the observed AUC value from the non-permuted data. The number of AUC values greater than the original value is the exact p-value for the best performing model (Model 1). In this case, the p-value of the best performing model is <0.01.

Final protein families for this subgroup (n = 30; 15 unique species) included PLA_2_, svMP, WAP/KUN, and BPP. Model selection produced equally performing models for the top 5 models, where AICc, pseudo-R^2^, and AUC were all equal (Fig 3A). For this reason, we recognized Model 1 as the best model (Fig 3C; p < 0.01), which had an accuracy of 93.3% (Fig 3B). False negatives were the only misclassifications produced by the model. Given this model’s high accuracy and overall effective discrimination of antibiotic efficacy, we consider this model to be performing well, recognizing the potential for Models 2 and 3 to also be equally accurate and valid models. Post-hoc accuracy analysis suggested that the data may not be overfit to our data subset (p = 0.23)

#### 3.2.2. Gram-positive facultative anaerobic coccus bacteria

For this subgroup (n = 34; 15 unique species), our final predictive protein families included PLA_2_, BPP, and DIS. Model selection demonstrated the null model as the highest performing model by AICc alone (S5A Fig). Model 2, however, outperformed the null model with respect to pseudo-R^2^ and AUC (S5A Fig). Post-hoc permutations for Model 2 revealed this model as not significant (S5C Fig; p = 0.258), despite a high accuracy of 76.5% (S5B Fig). Given the non-significant AUC value, we consider this model to perform poorly.

#### 3.2.3. Gram-positive anaerobic bacillus bacteria

**Fig 4:**
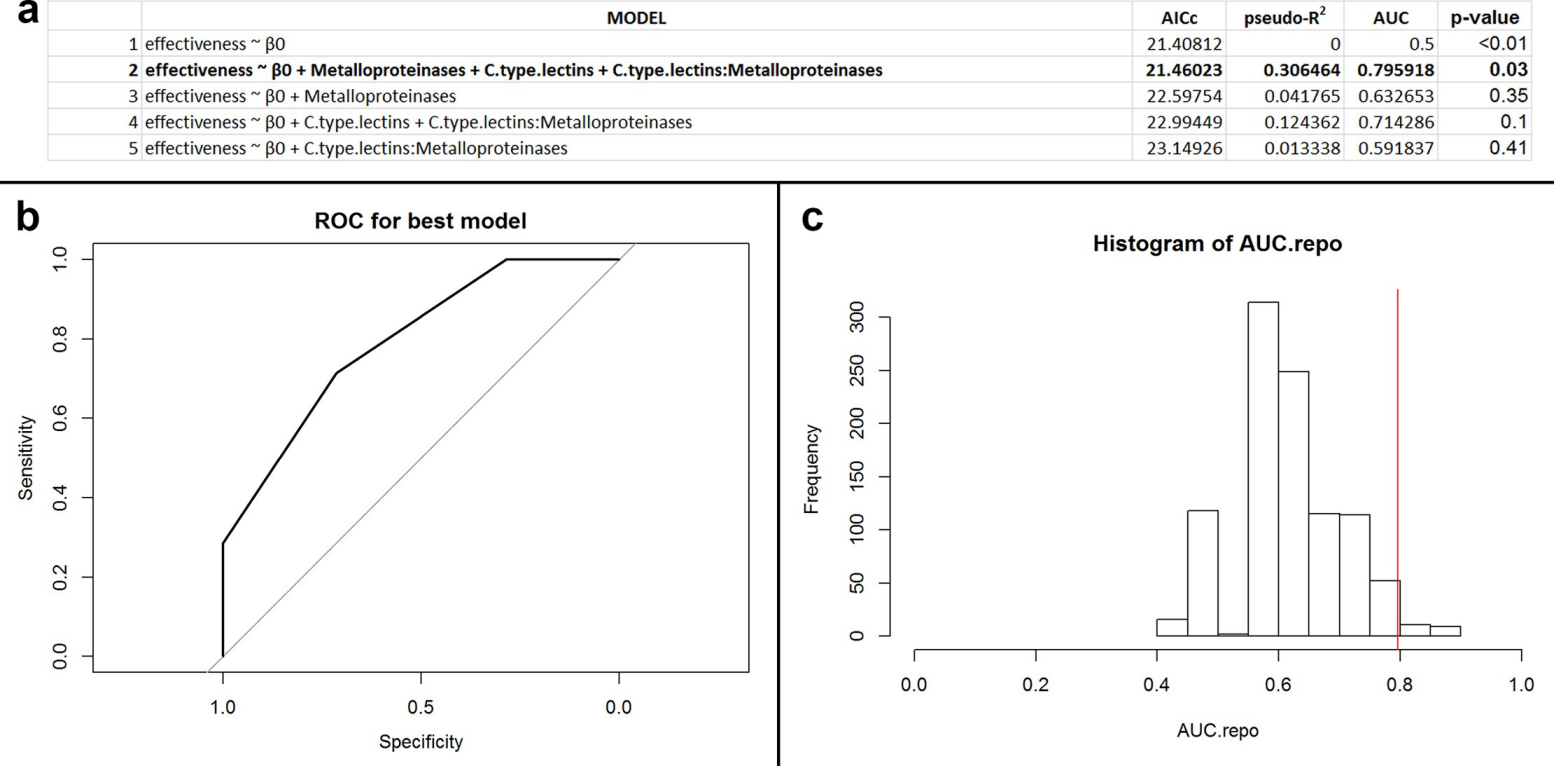
(a) Table of performances for the best five models that predict antibacterial efficacy using svMPs and CTLs for Viperidae snakes against Gram-positive, anaerobic, bacillus bacteria. The variable β_0_ indicates the intercept value for the model. Models defined as ‘effectiveness ~ β_0_’ are intercept models. Model 2, the best performing model, is in bold. (b) Receiver operator characteristic (ROC) curve for best performing model (Model 2). The area under the curve (AUC) of the ROC informs goodness of fit, where a value greater than 0.5 indicates the model performs better than random. In this instance, the AUC of the best performing model is 0.79. (c) Histogram of recalculated AUC values from permuted data. The red vertical line indicates the position of the observed AUC value from the non-permuted data. The number of AUC values greater than the original value is the exact p-value for the best performing model (Model 2). In this case, the p-value of the best performing model is 0.03.

For this subset (n = 14; 4 unique species), svMP and CTL were utilized for model selection. The null model was the best performing model by solely AICc (Fig 4A). The second best model, however, has a similar AICc with higher pseudo-R^2^ and AUC (Fig 4A). Model 2 also had a high accuracy rate (Fig 4B; 71.4%) and AUC permutation was significant (Fig 4C; p = 0.026). Together, these results demonstrate that Model 2 effectively discriminates antibiotic efficacy, and we consider this model to be performing well. Post-hoc accuracy analysis suggested that this data subset may be overfit to our data subset (p = 0.04).

#### 3.2.4. Gram-negative facultative anaerobic bacillus bacteria

This data subset (n = 47; 15 unique species) implemented PLA_2_ and WAP/KUN. Following model selection, Model 1 was the most competitive model by AICc, pseudo-R^2^, and AUC (S6A Fig). Although Model 1 was significant (S6C Fig; p = 0.026), it produced a high rate of false positives, resulting in low accuracy (S6B Fig; 63.8%). For these reasons, this model is considered a poorly performing model.

#### 3.2.5. Gram-negative aerobic bacillus bacteria

**Fig 5:**
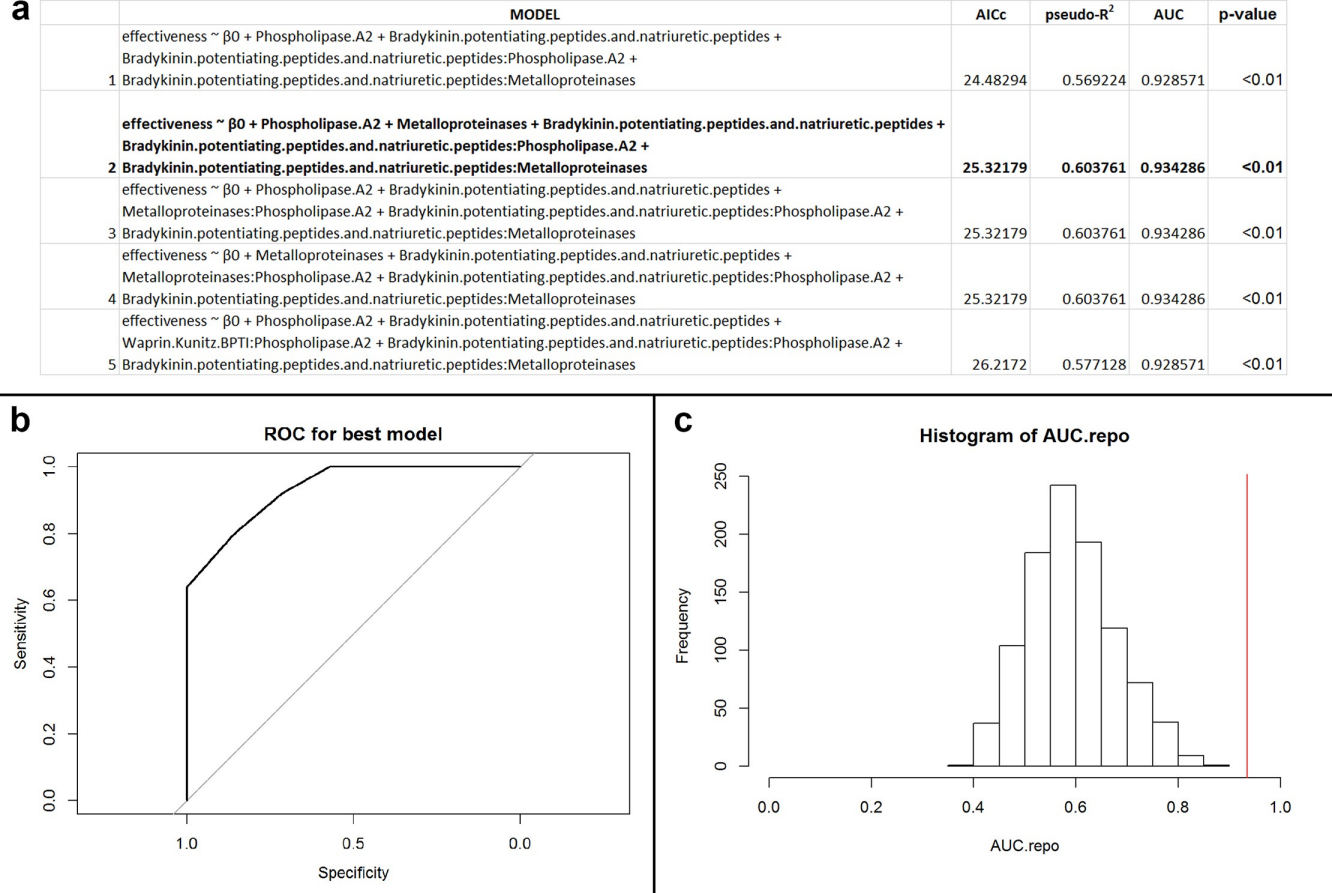
(a) Table of performances for the best five models that predict antibacterial efficacy using PLA_2_s, svMPs, BPPs, and WAP/KUNs for Viperidae snakes against Gram-negative, aerobic, bacillus bacteria. The variable β_0_ indicates the intercept value for the model. Models defined as ‘effectiveness ~ β_0_’ are intercept models. Model 2, the best performing model, is in bold. (b) Receiver operator characteristic (ROC) curve for best performing model (Model 2). The area under the curve (AUC) of the ROC informs goodness of fit, where a value greater than 0.5 indicates the model performs better than random. In this instance, the AUC of the best performing model is 0.93. (c) Histogram of recalculated AUC values from permuted data. The red vertical line indicates the position of the observed AUC value from the non-permuted data. The number of AUC values greater than the original value is the exact p-value for the best performing model (Model 2). In this case, the p-value of the best performing model is <0.01.

For this Viperid subset (n = 32; 14 unique species), predictive protein families for model selection include PLA_2_, svMP, WAP/KUN, and BPP. Despite Model 1 having the most competitive AICc following model selection, Model 2 was employed as the best model because of its superior pseudo-R^2^ as well as AUC values (Fig 5A). Model 2 is also highly significant (Fig 5C; p < 0.01) and has a high accuracy of 90.6% (Fig 5B). Despite its high accuracy, Model 2 does tend to produce a high rate of false positives with three of the seven ineffective venoms predicted as effective. Despite its high rate of false positives, we consider Model 2 a well performing model due to its high accuracy and high significance. Post-hoc accuracy analysis suggested that the model was not overfit to our data subset (p = 0.39).

### 3.3. Predicting efficacies for unexplored species

By applying our best performing and cross-validated models, we identified 6 Elapidae species with unknown efficacy as potentially effective against Gram-positive facultative anaerobic coccus bacteria as well as 6 Elapidae species that may be effective against Gram-negative facultative anaerobic bacillus bacteria (S1 Table). Likewise, we identified 15 Viperidae species that may be effective against Gram-positive facultative anaerobic bacillus bacteria, 34 against Gram-negative aerobic bacillus bacteria, and 32 against Gram-positive anaerobic bacillus bacteria (S2 Table). Although meeting our criteria for high model performance, the model for Viperidae against Gram-positive anaerobic bacillus bacteria should be considered with skepticism, as holdout cross-validation have shown this model’s accuracy to be highly sensitive to strong variance.

When well performing models were applied to Viperidae models, only *Dispholidus typus* was predicted as effective against Gram-negative facultative anaerobic bacillus as well as Gram-positive anaerobic bacillus bacteria (S3 Table). In contrast, the Elapidae models predicted all three Colubridae species as potentially effective against Gram-positive facultative anaerobic coccus as well as Gram-negative, facultative anaerobic, bacillus bacteria. Both Elapidae models had notable high accuracies with false positive rates of only 13% for each model. Likewise Viperidae models produced low false negative rates, ranging from 0–6.7%, giving credibility that the predicted negative efficacies are legitimate. Additionally, Viperidae models had false positive rates up to 32%. Together these false result rates suggest that predicted Colubrid efficacies are likely accurate and further supports the hypothesis that Colubrid venoms may yield positive results in antibacterial efficacy tests.

## Discussion

### 4.1. Model performance

Our results suggest that proteomic compositions are accurate predictors of snake venom’s antibacterial effectiveness, depending on snake family and bacterial classes. Models demonstrated greater accuracy in discriminating effectiveness when bacteria were grouped by Gram stain, morphology, and respiration strategy before being compared, which likely relates to the fact that the exact mechanisms guiding antibacterial effectiveness are complex. Despite these complexities and the underlying assumption that various proteoforms of an individual protein family have similar cytotoxic effects [28,29,30], our analyses generated several well performing statistical models and evidence opportunities for future research of snake venom’s antibacterial properties. The Elapidae models were accurate at predicting effectiveness against Gram-positive facultative anaerobic coccus bacteria as well as Gram-negative facultative anaerobic bacillus bacteria. Viperidae models accurately predicted effectiveness against Gram-positive facultative anaerobic bacillus, Gram-positive anaerobic bacillus, and Gram-negative aerobic bacillus bacteria. Previous studies have demonstrated the effectiveness of venom against all classes of bacteria for both the Elapidae [3,9,31] and the Viperidae [3,9,32,33], suggesting that crude venoms as well as isolated venom components are overall effective antibacterials.

Although some models performed well, false positives and false negatives did occur. For example, in tests against Gram-positive facultative anaerobic coccus bacteria, false positives were generated for *Naja haje*, *Naja melanoleuca*, and *Naja naja*. These false positives may be the result of absent or low values of WAP/KUN type inhibitors and high values of PLA_2_. Although the false positive may have been generated due to the high PLA_2_ content, the venom might be ineffective due to the absence of a WAP/KUN synergistic effect. Additionally, there are instances of false negatives in which an active component may be present but not in high enough concentrations such as *Daboia russelli* against Gram-negative facultative anaerobic bacillus bacteria. This false negative may have been generated due to a moderately low value of PLA_2_ [29] despite its known antibacterial activity [28,34,35]. Together, our results highlight that antibacterial effectiveness is a complex process with numerous potential requirements for individual protein families as well as their interactions.

### 4.2. Predictions vs mechanisms

Aside from their predictive value, it is crucial to consider the underlying antibacterial mechanisms of protein components individually as well as their functional interactions. Although specific proteins within a protein family may differ between venoms, they exhibit similar cytotoxic activities [28,36–38]. For all Elapidae, our analyses suggest PLA_2_, svMP, and WAP/KUN as the best predictors for antibacterial effectiveness. These protein families are all known to destabilize bacterial membranes [28,39]. Although PLA_2_, svMP, and WAP/KUN were the best predictive protein families, the biologically relevant activity of other venom components is worth noting. For example, 3FTXs can disrupt membrane integrity by interacting with the lipids in *S*. *aureus* and other Gram-positive bacteria [40–43]. Despite its apparent antibacterial capacities, 3FTX was a poor predictor of antibacterial effectiveness because its concentrations were correlated with other proteins and marginally varied between species.

For the Viperidae, antibacterial activity may be related to high level expression of a single component (i.e. PLA_2_) or compensation from other venom constituents (e.g. LAAO and svMP). Antibacterial effectiveness was associated with PLA_2_ concentration, a protein family with demonstrated activity against the lipid membranes of bacteria [28]. However, the absence or decreased expression of PLA_2_ in some species may be supplemented by other venom components that also exert antibacterial activity on the cell wall, such as LAAO [44] and svMP [45]. Irrespective of the relative abundance of each of these components, it is clear that their expression and direct activity against bacterial membranes is predictive of their antibacterial effectiveness. Moreover, understanding antibacterial activity is dependent upon gaining insights into the interplay between venom components.

### 4.3. Future perspectives

Our models suggest that certain snake families may be better at targeting a given bacterial class over the others. The strength of these models is that they provide data driven *a priori* hypotheses to be tested by highlighting snakes and bacterial classes of interest based on predicted efficacy. There are numerous species of Elapidae for which venom composition exists but have not been tested for effectiveness as an antibacterial agent (S2 Table). These species include but are not limited to: *Calliophis bivirgata* [46], *Hydrophis schistosus* [47], *Micrurus dumerilii* [48], *Micrurus mosquitensis* [49] and *Naja atra* [50]. As with the Elapidae, there are several species of Viperidae with venom composition data but no efficacy data (S3 Table). These species include but are not limited to: *Bothriechis aurifer* [51], *Crotalus tigris* [52], *Hypnale hypnale* [53], *Porthidium nasutum* [54], and *Sistrurus catenatus* [55,56].

Aside from the Viperidae and Elapidae, Colubridae venom offers a unique opportunity to study conserved venom constituents as well as neofunctionalized protein families with the potential for exploring antibacterial activity [4,57]. Previous studies have shown that bioactive constituents of venom evolved early within the Colubroidea (clade containing viperids, elapids, and colubrids) and many protein families found within the highly toxic elapids and viperids may also be found in the colubrids [58,59]. Despite previous researchers highlighting the potential for colubrids as novel sources of antibacterial components [5,60], research concerning this group of snakes is limited. To date, only three studies have reported the complete proteome of colubrid species [61–63] and empirical evidence of antibacterial efficacy is based on three different species [3,64] which have resulted in inconclusive results. Inputting colubrid species with known venom proteomes into the well performing viperid models suggests that, although two of the three would be ineffective against Gram-negative facultative anaerobic bacillus, Gram-negative aerobic bacillus, and Gram-positive anaerobic bacillus bacteria (S4 Table), one, *Dispholidus typus* would be effective against Gram-negative facultative anaerobic bacillus bacteria (although it may be a false positive due to high PLA_2_ concentration).

Additional work concerning Colubridae venom composition and antibacterial efficacy are needed to refine our models, and such a shortage of available data presents multiple avenues for subsequent investigations. Furthermore, the continuous addition of antibacterial efficacy and venom proteome data, including individual proteoforms, will provide opportunities for the model to expand and become more inclusive. Additional data may provide more robust accuracies in individual models instead of using combinatory groupings as utilized in this study. Lastly, the addition of non-protein constituents including small organic molecules may enhance the accuracy of predictions to further elucidate potential mechanisms of action and predictive power.

## Supporting information

S1 Fig PERMANOVA table for Elapidae venom as predicted by snake species as well as boxplot of venom protein composition for each species. Because of potential for intraspecific variation in snake venom proteomic composition to exceed interspecific variation, we performed a PERMANOVA, with all protein families as a multivariate response of snake species. For the Elapidae species, visual inspection of associated boxplots as well as PERMANOVA results (p = 0.002) confirm that indeed variation between species is greater than within species.
(TIF)

S2 Fig PERMANOVA table for Viperidae venom as predicted by snake species as well as boxplot of venom protein composition for each species. Because of potential for intraspecific variation in snake venom proteomic composition to exceed interspecific variation, we performed a PERMANOVA, with all protein families as a multivariate response of snake species. For the Viperidae species, visual inspection of associated boxplots as well as PERMANOVA results (p = 0.001) confirm that indeed variation between species is greater than within species.
(TIF)

S3 Fig (a) Table of performances for the best five models that predict antibacterial efficacy using PLA_2_s, svMPs, and WAP/KUNs for Elapidae snakes against Gram-positive, facultative anaerobic, bacillus bacteria. The variable β_0_ indicates the intercept value for the model. Models defined as ‘effectiveness ~ β_0_’ are intercept models. Model 2, the best performing model, is in bold. (b) Receiver operator characteristic (ROC) curve for best performing model (Model 2). The area under the curve (AUC) of the ROC informs goodness of fit, where a value greater than 0.5 indicates the model performs better than random. In this instance, the AUC of the best performing model is 0.62. (c) Histogram of recalculated AUC values from permuted data. The red vertical line indicates the position of the observed AUC value from the non-permuted data. The number of AUC values greater than the original value is the exact p-value for the best performing model (Model 2). In this case, the p-value of the best performing model is 0.27.
(TIF)

S4 Fig (a) Table of performances for the best five models that predict antibacterial efficacy using PLA_2_s, svMPs, and WAP/KUNs for Elapidae snakes against Gram-negative, aerobic, bacillus bacteria. The variable β_0_ indicates the intercept value for the model. Models defined as ‘effectiveness ~ β_0_’ are intercept models. Model 2, the best performing model, is in bold. (b) Receiver operator characteristic (ROC) curve for best performing model (Model 2). The area under the curve (AUC) of the ROC informs goodness of fit, where a value greater than 0.5 indicates the model performs better than random. In this instance, the AUC of the best performing model is 0.58. (c) Histogram of recalculated AUC values from permuted data. The red vertical line indicates the position of the observed AUC value from the non-permuted data. The number of AUC values greater than the original value is the exact p-value for the best performing model (Model 2). In this case, the p-value of the best performing model is 0.44.
(TIF)

S5 Fig (a) Table of performances for the best five models that predict antibacterial efficacy using PLA_2_s, svMPs, BPPs, and WAP/KUNs for Viperidae snakes against Gram-positive, facultative anaerobic, coccus bacteria. The variable β_0_ indicates the intercept value for the model. Models defined as ‘effectiveness ~ β_0_’ are intercept models. Model 2, the best performing model, is in bold. (b) Receiver operator characteristic (ROC) curve for best performing model (Model 2). The area under the curve (AUC) of the ROC informs goodness of fit, where a value greater than 0.5 indicates the model performs better than random. In this instance, the AUC of the best performing model is 0.63. (c) Histogram of recalculated AUC values from permuted data. The red vertical line indicates the position of the observed AUC value from the non-permuted data. The number of AUC values greater than the original value is the exact p-value for the best performing model (Model 2). In this case, the p-value of the best performing model is 0.26.
(TIF)

S6 Fig (a) Table of performances for the best five models that predict antibacterial efficacy using svMPs and WAP/KUNs for Viperidae snakes against Gram-negative, anaerobic, bacillus bacteria. The variable β_0_ indicates the intercept value for the model. Models defined as effectiveness ~ ‘β_0_’ are intercept models. Model 2, the best performing model, is in bold. (b) Receiver operator characteristic (ROC) curve for best performing model (Model 2). The area under the curve (AUC) of the ROC informs goodness of fit, where a value greater than 0.5 indicates the model performs better than random. In this instance, the AUC of the best performing model is 0.61. (c) Histogram of recalculated AUC values from permuted data. The red vertical line indicates the position of the observed AUC value from the non-permuted data. The number of AUC values greater than the original value is the exact p-value for the best performing model (Model 2). In this case, the p-value of the best performing model is <0.01.
(TIF)

S1 File Technical documentation for reproducing analysis. This html document details the R code used for this analysis. All analyses can be performed using the data provided on the Open Science Framework page for this project (28). Users wishing to recreate our analyses with their own data will need to name all column headers such that they are internally consisten with the R script as well as any data used from Meyer et al. (28).
(HTML)

S2 File Supplemental references for revision 2. (DOCX)

S1 Table Coefficients and standard errors for best fitted logistic models. (XLSX)

S2 Table Elapidae species with known venom proteomes and their predicted antibacterial activity. Bolded species indicate predicted effectiveness against the bacterial class.
(XLSX)

S3 Table Viperidae species with known venom proteomes and their predicted antibacterial activity. Bolded species indicate predicted effectiveness against the bacterial class.
(XLSX)

S4 Table Colubridae species with known venom proteomes and their predicted antibacterial activity. Antibacterial activity predictions were made using both the Elapidae and Viperidae models.
(XLSX)
