# Supplementary figures and images for: Predicting antibacterial activity from snake venom proteomes
Source: PLoS One. 2020 Jan 24;15(1):e0226807. doi: 10.1371/journal.pone.0226807 (PMC6980403; doi:10.1371/journal.pone.0226807)

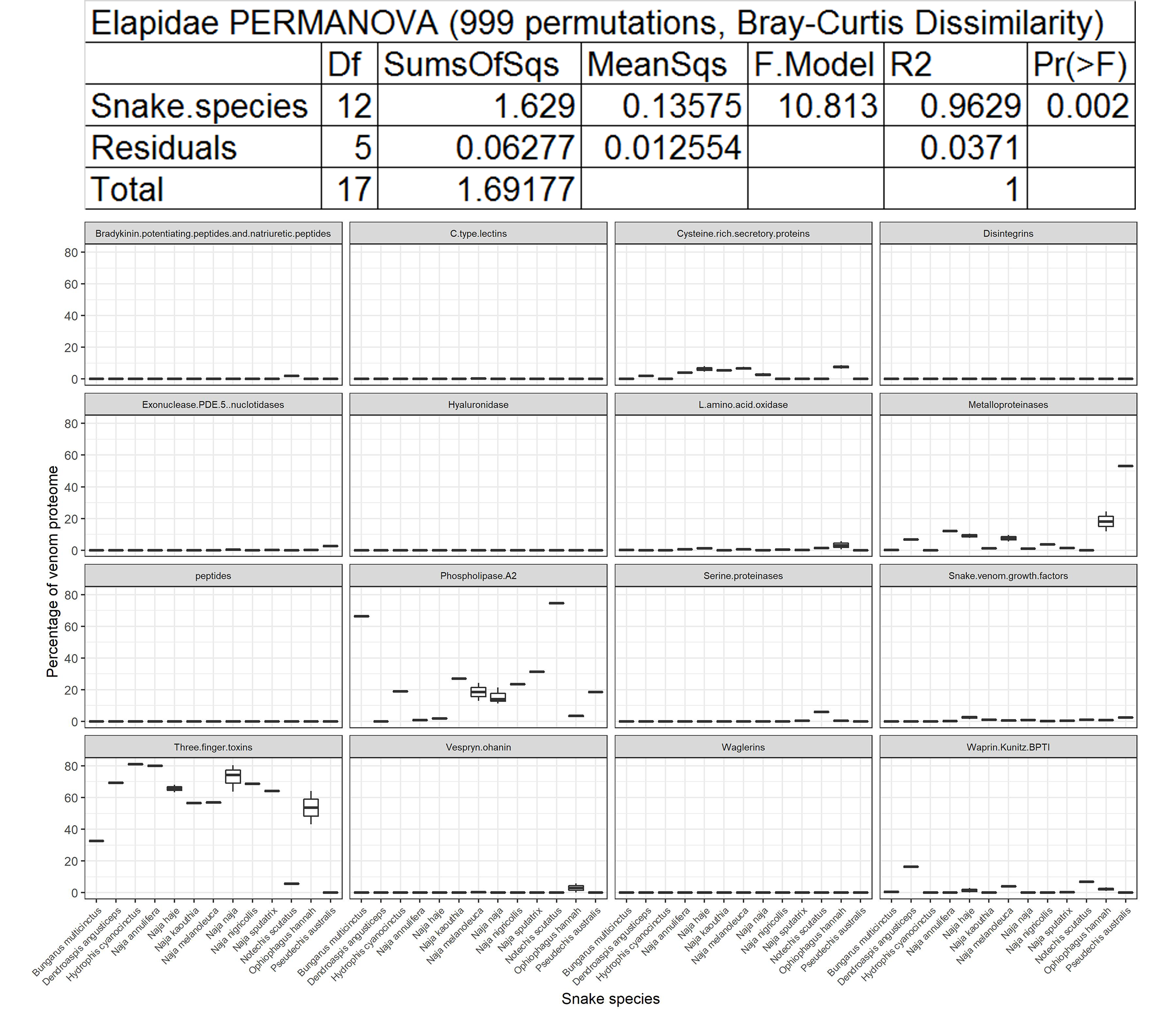

Supplement: S1 Fig — Because of potential for intraspecific variation in snake venom proteomic composition to exceed interspecific variation, we performed a PERMANOVA, with all protein families as a multivariate response of snake species. For the Elapidae species, visual inspection of associated boxplots as well as PERMANOVA results (p = 0.002) confirm that indeed variation between species is greater than within species. (TIF) [file pone.0226807.s001.tif]

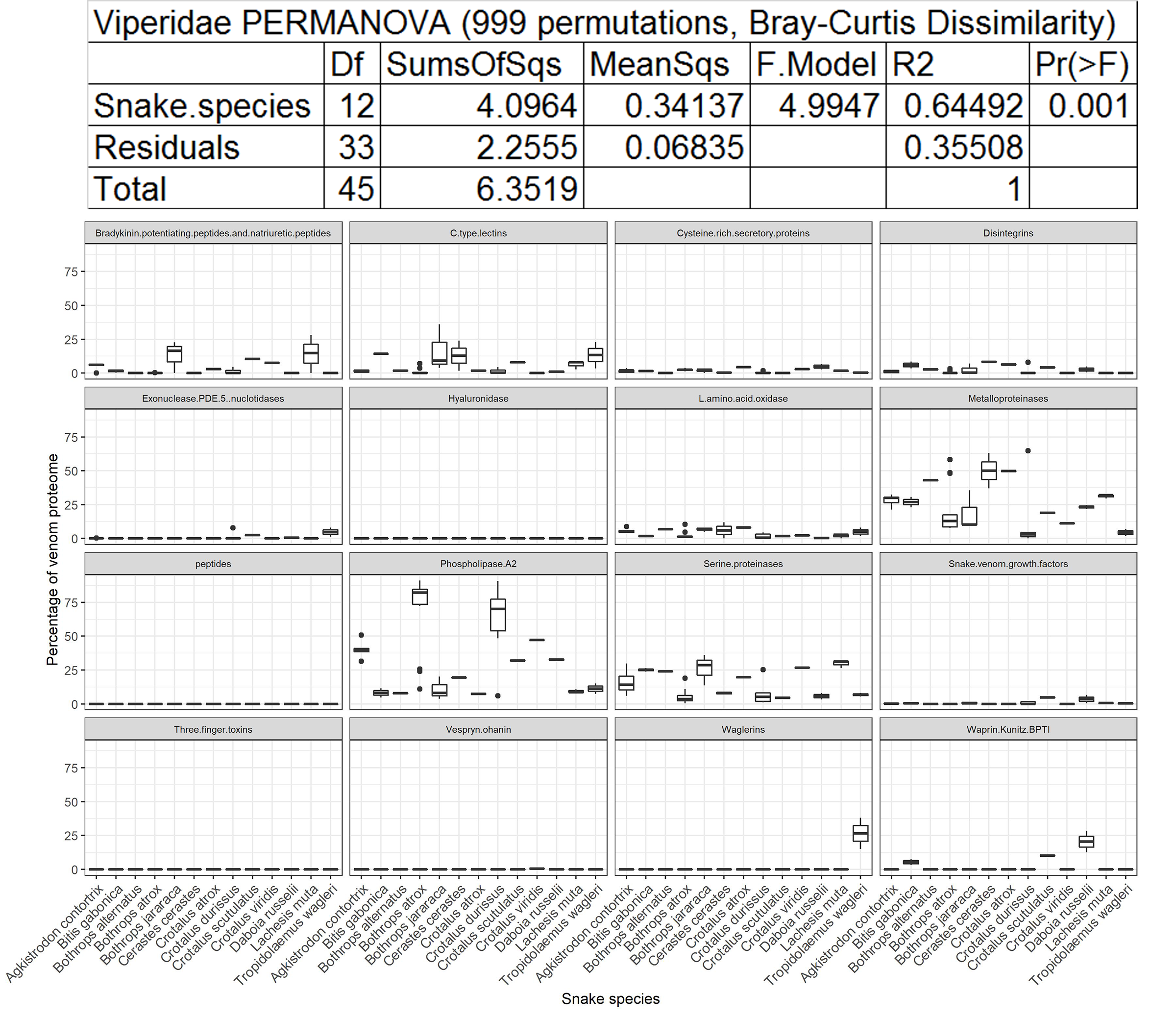

Supplement: S2 Fig — Because of potential for intraspecific variation in snake venom proteomic composition to exceed interspecific variation, we performed a PERMANOVA, with all protein families as a multivariate response of snake species. For the Viperidae species, visual inspection of associated boxplots as well as PERMANOVA results (p = 0.001) confirm that indeed variation between species is greater than within species. (TIF) [file pone.0226807.s002.tif]

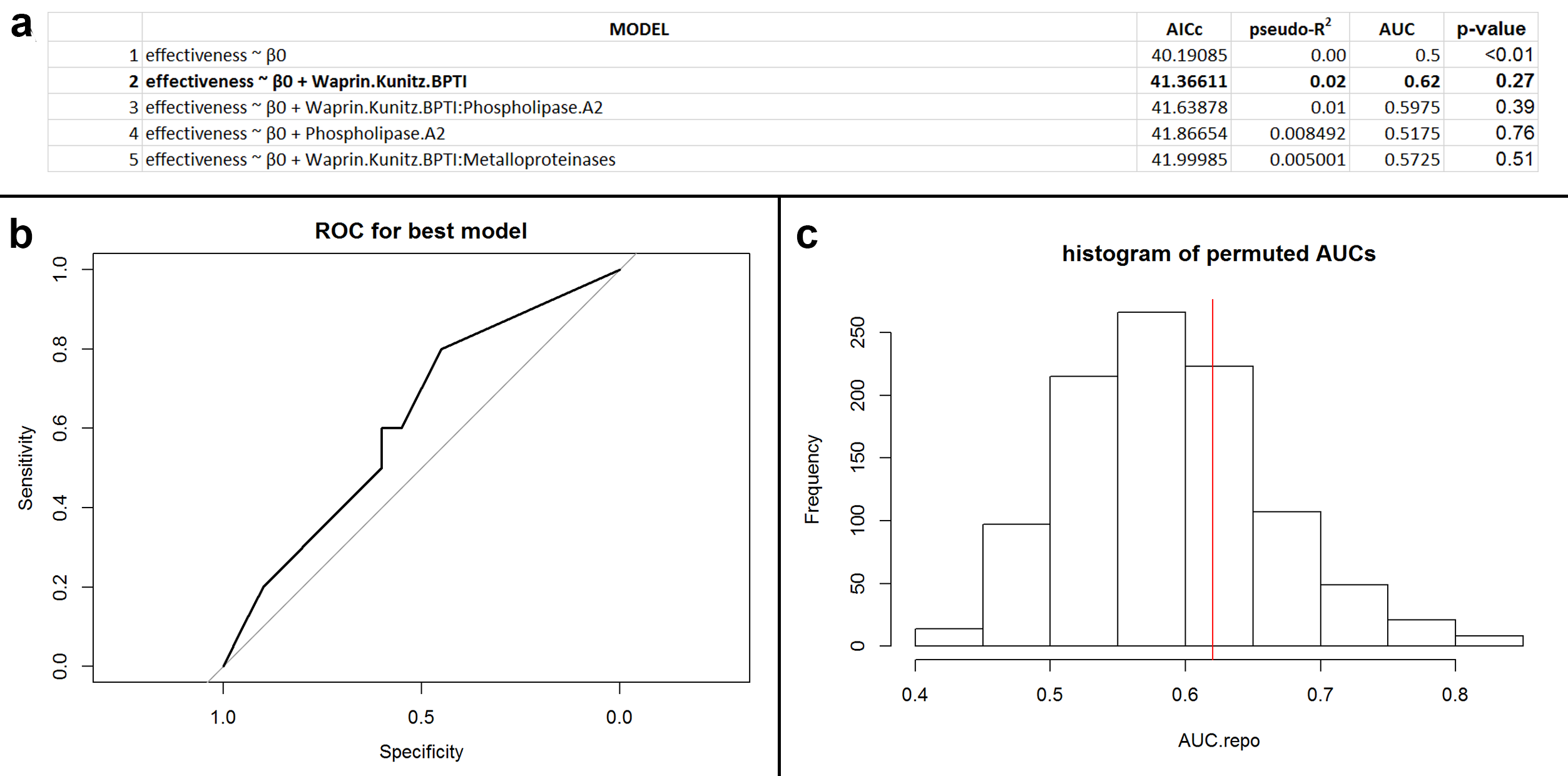

Supplement: S3 Fig — (a) Table of performances for the best five models that predict antibacterial efficacy using PLA2s, svMPs, and WAP/KUNs for Elapidae snakes against Gram-positive, facultative anaerobic, bacillus bacteria. The variable β0 indicates the intercept value for the model. Models defined as ‘effectiveness ~ β0’ are intercept models. Model 2, the best performing model, is in bold. (b) Receiver operator characteristic (ROC) curve for best performing model (Model 2). The area under the curve (AUC) of the ROC informs goodness of fit, where a value greater than 0.5 indicates the model performs better than random. In this instance, the AUC of the best performing model is 0.62. (c) Histogram of recalculated AUC values from permuted data. The red vertical line indicates the position of the observed AUC value from the non-permuted data. The number of AUC values greater than the original value is the exact p-value for the best performing model (Model 2). In this case, the p-value of the best performing model is 0.27. (TIF) [file pone.0226807.s003.tif]

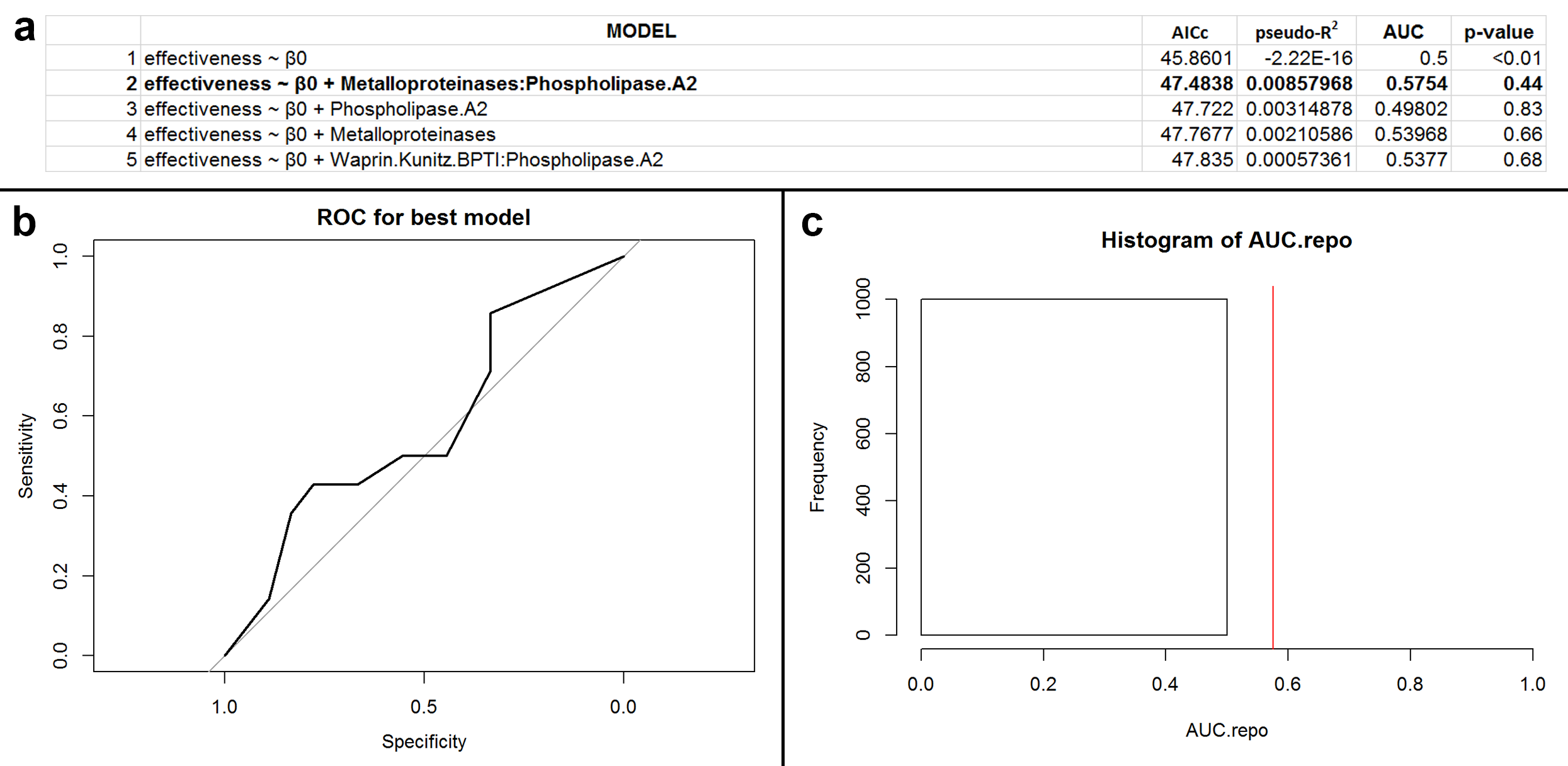

Supplement: S4 Fig — (a) Table of performances for the best five models that predict antibacterial efficacy using PLA2s, svMPs, and WAP/KUNs for Elapidae snakes against Gram-negative, aerobic, bacillus bacteria. The variable β0 indicates the intercept value for the model. Models defined as ‘effectiveness ~ β0’ are intercept models. Model 2, the best performing model, is in bold. (b) Receiver operator characteristic (ROC) curve for best performing model (Model 2). The area under the curve (AUC) of the ROC informs goodness of fit, where a value greater than 0.5 indicates the model performs better than random. In this instance, the AUC of the best performing model is 0.58. (c) Histogram of recalculated AUC values from permuted data. The red vertical line indicates the position of the observed AUC value from the non-permuted data. The number of AUC values greater than the original value is the exact p-value for the best performing model (Model 2). In this case, the p-value of the best performing model is 0.44. (TIF) [file pone.0226807.s004.tif]

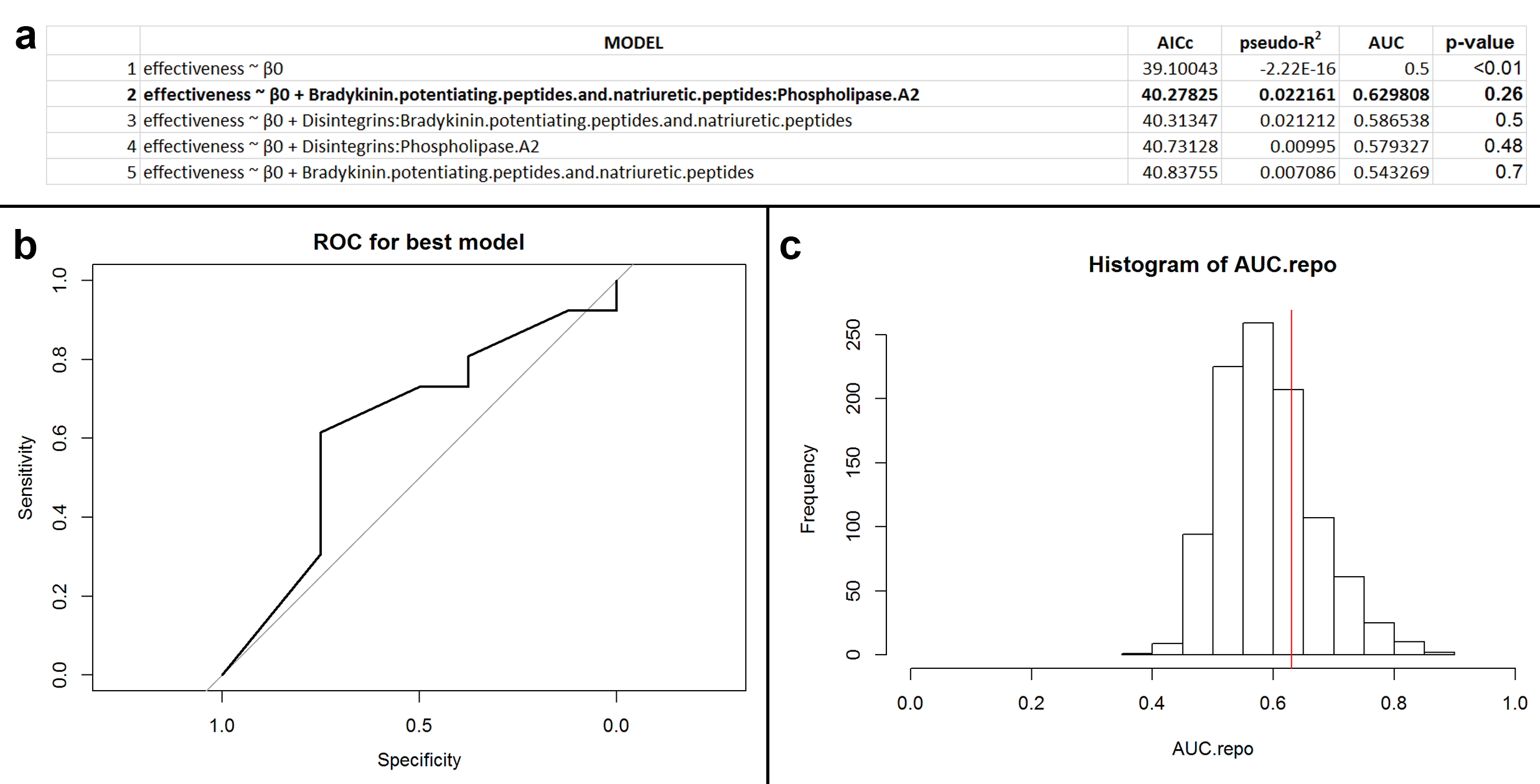

Supplement: S5 Fig — (a) Table of performances for the best five models that predict antibacterial efficacy using PLA2s, svMPs, BPPs, and WAP/KUNs for Viperidae snakes against Gram-positive, facultative anaerobic, coccus bacteria. The variable β0 indicates the intercept value for the model. Models defined as ‘effectiveness ~ β0’ are intercept models. Model 2, the best performing model, is in bold. (b) Receiver operator characteristic (ROC) curve for best performing model (Model 2). The area under the curve (AUC) of the ROC informs goodness of fit, where a value greater than 0.5 indicates the model performs better than random. In this instance, the AUC of the best performing model is 0.63. (c) Histogram of recalculated AUC values from permuted data. The red vertical line indicates the position of the observed AUC value from the non-permuted data. The number of AUC values greater than the original value is the exact p-value for the best performing model (Model 2). In this case, the p-value of the best performing model is 0.26. (TIF) [file pone.0226807.s005.tif]

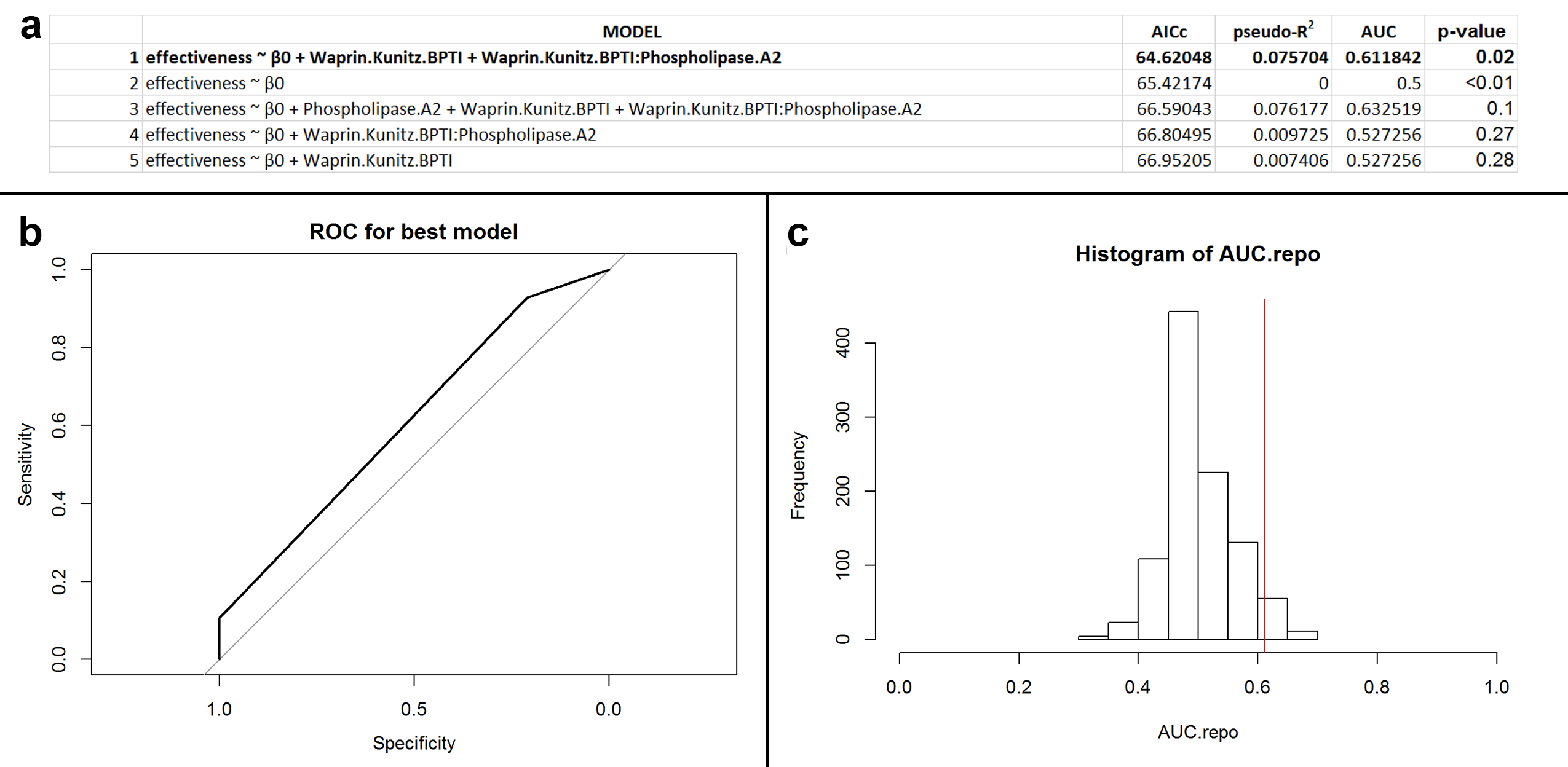

Supplement: S6 Fig — (a) Table of performances for the best five models that predict antibacterial efficacy using svMPs and WAP/KUNs for Viperidae snakes against Gram-negative, anaerobic, bacillus bacteria. The variable β0 indicates the intercept value for the model. Models defined as effectiveness ~ ‘β0’ are intercept models. Model 2, the best performing model, is in bold. (b) Receiver operator characteristic (ROC) curve for best performing model (Model 2). The area under the curve (AUC) of the ROC informs goodness of fit, where a value greater than 0.5 indicates the model performs better than random. In this instance, the AUC of the best performing model is 0.61. (c) Histogram of recalculated AUC values from permuted data. The red vertical line indicates the position of the observed AUC value from the non-permuted data. The number of AUC values greater than the original value is the exact p-value for the best performing model (Model 2). In this case, the p-value of the best performing model is <0.01. (TIF) [file pone.0226807.s006.tif]
