# Supplementary material for: Predicting antibacterial activity from snake venom proteomes
Source: PLoS One. 2020 Jan 24;15(1):e0226807. doi: 10.1371/journal.pone.0226807 (PMC6980403; doi:10.1371/journal.pone.0226807)
Supplement: S1 File — This html document details the R code used for this analysis. All analyses can be performed using the data provided on the Open Science Framework page for this project (28). Users wishing to recreate our analyses with their own data will need to name all column headers such that they are internally consisten with the R script as well as any data used from Meyer et al. (28). (HTML) [file pone.0226807.s007.html]

Supplemental Information


# Supplemental Information

#### *Justin L. Rheubert, Michael F. Meyer, Raeshelle M. Strobel, Megan A. Pasternak, Robert A. Charvat*

#### *29 August 2018*

# For the reader

This is a supplemental technical document and tutorial for model selection and cross-validation techniques using the R statistical environment to accompany the manuscript Rheubert et al. (2018).

Data collection was conducted by Justin L. Rheubert (University of Findlay, Findlay, OH 45840). Statistical analyses in this vignette were conducted by Michael F. Meyer (Washington State University, Pullman, WA 99164).

This work can be cited as:

Rheubert, J.L., Meyer, M.F., Strobel, R.M., Pasternak, M.A., & R.A. Charvart. 2018. Predicting antibacterial activity from snake venom proteomes.

For more information regarding this project or its citation, please contact either Justin L. Rheubert (rheubert@findlay.edu) or Michael F. Meyer (michael.f.meyer@wsu.edu).

# Packages required

The following packages are required:

1. Data Manipluation: tidyr, dplyr, stringr
2. Model Selection and Statistical Operations: car, MASS, glmulti, pROC

```
# This section loads required packages and creates a function
# to builds a pairs plot matrix with significance values on
# the off-daigonals. This section can be copy and pasted as
# is, assuming the user has the below packages installed on
# their machine.

library(tidyr)  #Required for data manipulation
library(dplyr)  #Required for data manipulation
library(stringr)  #Required for data manipulation
library(car)  #Required for pairs plots
library(MASS)  #Required for pairs plots
library(glmulti)  #Required for model selection
library(pROC)  #Required for model selection

# Generic function use to create correlation pairs plots with
# significant correlations on the upper panel off-diagonals
# and plots of correlations on the low panel off-diagonals

panel.cor <- function(x, y, digits = 2, cex.cor, ...) {
    usr <- par("usr")
    on.exit(par(usr))
    par(usr = c(0, 1, 0, 1))
    # correlation coefficient
    r <- cor(x, y)
    txt <- format(c(r, 0.123456789), digits = digits)[1]
    txt <- paste("r= ", txt, sep = "")
    text(0.5, 0.6, txt)
    
    # p-value calculation
    p <- cor.test(x, y)$p.value
    txt2 <- format(c(p, 0.123456789), digits = digits)[1]
    txt2 <- paste("p= ", txt2, sep = "")
    if (p < 0.01) 
        txt2 <- paste("p= ", "<0.01", sep = "")
    text(0.5, 0.4, txt2)
}
```

# Data import and description

First, we imported the data into the R environment. Note that this data subset is specifically composed of Elapidae species that were tested against gram negative, facultative anaerobic, bacillus bacteria.

```
#' Load data CSV
#' The variable name 'elap.gramneg.fac.bacillus' can be changed
#' for future use. 
elap.gramneg.fac.bacillus <- read.csv("elap.gramneg.fac.bacillus.csv", 
    header = TRUE)
```

- **Snake.species** and **Family**: Genus and Species of snake, as well as Family of Snake (All should be Elapidae)
- **Three.finger.toxins, Phospholipase.A2, Metalloproteinases, Serine.proteinases, Disintegrins, C.type.lectins, peptides, Vespryn.ohanin, Exonuclease.PDE.5..nuclotidases, Waglerins, Cysteine.rich.secretory.proteins, Snake.venom.growth.factors, L.amino.acid.oxidase, Waprin.Kunitz.BPTI, Hyaluronidase**: Arcsine transformed protein proportions.
- **Toxin**: Type of toxin considered from original study (Should be Crude\_Venom)
- **bacteria**: Bacterial species considered in the study
- **effectiveness**: Whether or not crude venom was effective as an antibiotic. Binary responses coded as 0 for ineffective and 1 as effective.
- **Respiration**, **Morphology**, and **Gram.Stain**: Metadata on the bacteria of study. (Should be Facultative anaerobic, bacillus, and gram negative respectively)

# Summary statistics

In order to identify proteins with the greatest predictive power, we compared interspecific mean and variance of venom protein components. Criterions for protein selection included.

1. Variance needs to be greater than 0.01.
2. Variance to Mean Ratio needs to be greater than or equal to 0.05, meaning that the variance is at least 5% of the mean.

```
#' This section creates a data frame of the interspecific mean, variance, 
#' and variance/mean ratio for all proteins. 
#' Assuming a similar analysis, the user may wish to change: 
#' 1. The number of proteins considered (Currently 16)


# Creates a vector of interspecific mean percentages
mean <- as.vector(sapply(elap.gramneg.fac.bacillus[, 3:18], mean))
# Creates a vector of interspecific variances
var <- as.vector(sapply(elap.gramneg.fac.bacillus[, 3:18], var))
# Combines those mean and variance into one dataframe
mean.var.elap <- data.frame(cbind(mean[1:16], var[1:16]))
# Renames columns as mean and variance
colnames(mean.var.elap)[colnames(mean.var.elap) == "X1"] <- "Mean"
colnames(mean.var.elap)[colnames(mean.var.elap) == "X2"] <- "Variance"
# Calculates the variance:mean ratio
mean.var.elap <- dplyr::mutate(mean.var.elap, Var.Mean.RATIO = Variance/Mean)
# Adds rownames, such that row names are protein families
row.names(mean.var.elap) <- colnames(elap.gramneg.fac.bacillus[, 3:18])
```

|  | Mean | Variance | Var.Mean.RATIO |
| --- | --- | --- | --- |
| Three.finger.toxins | 0.8113154 | 0.1029969 | 0.1269505 |
| Phospholipase.A2 | 0.3975931 | 0.0646446 | 0.1625899 |
| Metalloproteinases | 0.2654288 | 0.0498894 | 0.1879579 |
| Waprin.Kunitz.BPTI | 0.1096811 | 0.0138297 | 0.1260897 |
| L.amino.acid.oxidase | 0.1439121 | 0.0133742 | 0.0929328 |
| Cysteine.rich.secretory.proteins | 0.0656119 | 0.0030976 | 0.0472111 |
| Serine.proteinases | 0.0242164 | 0.0029264 | 0.1208433 |
| Vespryn.ohanin | 0.0319233 | 0.0029112 | 0.0911924 |
| Snake.venom.growth.factors | 0.0802526 | 0.0028766 | 0.0358440 |
| Exonuclease.PDE.5..nuclotidases | 0.0279614 | 0.0022487 | 0.0804231 |
| C.type.lectins | 0.0076465 | 0.0003691 | 0.0482759 |
| peptides | 0.0011394 | 0.0000272 | 0.0239141 |
| Family | NA | 0.0000000 | NA |
| Disintegrins | 0.0000000 | 0.0000000 | NaN |
| Waglerins | 0.0000000 | 0.0000000 | NaN |
| Hyaluronidase | 0.0000000 | 0.0000000 | NaN |

Three-Finger toxins, Phospholipase A2, Metalloproteinases, Waprin-Kunitz,and L-Amino-Acid Oxidases meet our variance criteria. These are the proteins that we will use for predictive measures.

Prior to model generation, though, we examine for cross-correlation between proteins, and remove proteins that covary (i.e., p < 0.01).

We eliminate Three Finger Toxins and L-Amino-Acid-Oxidases because they are strongly correlated Phospholipases, Waprin Kunitz BPTI and Metalloproteinases.

# Model selection and cross-validation

Our approach to model selection can be summarized as an exhaustive model selection approach, using AICc, pseudo-\(R^2\), and AUC cross-model comparison metrics, and then validation through a non-parametric, permutation of AUC values for the best performing model.

## Exhaustive model generation

First, we employed the glmulti package to generate all possible model combinations, including potential interactions between predictors. Models are ordered by increasing AICc.

```
#' This section performs the exhaustive model selection, and creates a S4 object where 
#' the results of the top 100 best performing models are stored. 
#' Assuming a similar analysis, the user may wish to change: 
#' 1. The model response and predictors
#' 2. Whether interactions are to be considered (Currently yes)
#' 3. Exhaustive or non-exhaustive approach (Currently yes)
#' 4. Information criterion (Currently AICc)
#' 5. Number of models retained (Currently 100)
#' 6. Type of model fit (Currently glm)
#' 7. Family of model fit (Currently binomial)


elap.gramneg.fac.bacillus.model <-
  glmulti(effectiveness ~  Phospholipase.A2 + Metalloproteinases + Waprin.Kunitz.BPTI, 
          data = elap.gramneg.fac.bacillus,
          level = 2,               # Interactions considered
          method = "h",            # Exhaustive approach
          crit = "aic",            # AIC as criteria
          confsetsize = 100,       # Keep 100 best models
          plotty = T, report =T,   # Plot and give interim reports
          fitfunction = "glm",     # Fit using a glm
          family = binomial)       # Binomial fits a logistic binomial
```

Next, we calculated pseudo-\(R^2\) and AUC to assess data fit. While there are many definitions of pseudo-\(R^2\), we used the McFadden definition, as \(R^2 = 1 - \frac{Null Deviance}{Residual Deviance}\). We also calculated AUC for each model, using each model’s Receiver Operator Characteristic (ROC) curve.

## Pseudo-\(R^2\) and AUC calculation

```
#' This section calculates and aggregates model criteria for each model generated.
#' Assuming a similar analysis, this section can be copied and pasted with no alterations. 

# First, create empty vectors for AIC, MODEL, AUC, and
# RSQUARED. These will need to have a length up to the number
# of formulas retain in the glmulti command (i.e., 100).
AIC <- rep(0, length(elap.gramneg.fac.bacillus.model@formulas))
MODEL <- rep(NA, length(elap.gramneg.fac.bacillus.model@formulas))
AUC <- rep(0, length(elap.gramneg.fac.bacillus.model@formulas))
RSQUARED <- rep(0, length(elap.gramneg.fac.bacillus.model@formulas))
# The for lopp below calculates AIC, AUC, pseudo-Rsquared and
# the model into the element of each vector created in the
# commands above.
for (i in 1:length(elap.gramneg.fac.bacillus.model@formulas)) {
    fit <- glm(paste(as.character(elap.gramneg.fac.bacillus.model@formulas[i])), 
        data = elap.gramneg.fac.bacillus, family = binomial)
    MODEL[i] <- paste(as.character(elap.gramneg.fac.bacillus.model@formulas[i]))
    AIC[i] <- fit$aic
    predictpr <- predict(fit, type = "response")
    ROC <- pROC::roc(elap.gramneg.fac.bacillus$effectiveness ~ 
        predictpr)
    AUC[i] <- pROC::auc(ROC)
    RSQUARED[i] <- 1 - (fit$deviance/fit$null.deviance)
}
# Create an idexing variable
INDEX <- seq(1:length(elap.gramneg.fac.bacillus.model@formulas))
# Combine INDEX, MODEL, AIC, RSQUARED, AND AUC into one
# dataframe
elap.gramneg.fac.bacillus.fits <- data.frame(INDEX, MODEL, AIC, 
    RSQUARED, AUC)
# Convert MODEL to a character
elap.gramneg.fac.bacillus.fits$MODEL <- as.character(elap.gramneg.fac.bacillus.fits$MODEL)
# Convert AIC, AUC, and pseudo-Rsquared to a numeric
elap.gramneg.fac.bacillus.fits$AIC <- as.numeric(elap.gramneg.fac.bacillus.fits$AIC)
elap.gramneg.fac.bacillus.fits$RSQUARED <- as.numeric(elap.gramneg.fac.bacillus.fits$RSQUARED)
elap.gramneg.fac.bacillus.fits$AUC <- as.numeric(elap.gramneg.fac.bacillus.fits$AUC)
```

| No. | Model | AIC | \(R^2\) | AUC |
| --- | --- | --- | --- | --- |
| 1 | effectiveness ~ 1 + Metalloproteinases + Waprin.Kunitz.BPTI:Phospholipase.A2 + Waprin.Kunitz.BPTI:Metalloproteinases | 44.99 | 0.37 | 0.86 |
| 2 | effectiveness ~ 1 + Metalloproteinases + Waprin.Kunitz.BPTI + Metalloproteinases:Phospholipase.A2 + Waprin.Kunitz.BPTI:Phospholipase.A2 + Waprin.Kunitz.BPTI:Metalloproteinases | 45.91 | 0.43 | 0.87 |
| 3 | effectiveness ~ 1 + Metalloproteinases + Metalloproteinases:Phospholipase.A2 + Waprin.Kunitz.BPTI:Phospholipase.A2 + Waprin.Kunitz.BPTI:Metalloproteinases | 46.29 | 0.39 | 0.86 |
| 4 | effectiveness ~ 1 + Phospholipase.A2 + Metalloproteinases + Waprin.Kunitz.BPTI:Phospholipase.A2 + Waprin.Kunitz.BPTI:Metalloproteinases | 46.97 | 0.37 | 0.86 |
| 5 | effectiveness ~ 1 + Metalloproteinases + Waprin.Kunitz.BPTI + Waprin.Kunitz.BPTI:Phospholipase.A2 + Waprin.Kunitz.BPTI:Metalloproteinases | 46.99 | 0.37 | 0.85 |
| 6 | effectiveness ~ 1 + Phospholipase.A2 + Metalloproteinases + Waprin.Kunitz.BPTI + Waprin.Kunitz.BPTI:Metalloproteinases | 47.18 | 0.37 | 0.85 |

Based on our model selection criteria, Model 2 is the best performing model. Although Model 1 has a better AICc, Model 2 has a better \(R^2\) and AUC.

## Confusion matrix and accuracy

Next, we assessed the ROC curve for the best performing model. To assess confusion within the model, we also isolated false results so as to understand similarities among misclassified snakes.

```
#' This section runs diagnostics on the best performing model. A full explanation of the 
#' model diagnostics are within the methods section of the manuscript. 
#' Assuming a similar analysis, the user may wish to change: 
#' 1. The best fit model (currently model 2)
#' 2. Threshold percentage for defining effective venom (currently > 50%)


# Fit a logistic regression for the best performing model
# (i.e., model 2).
fit <- glm(paste(as.character(elap.gramneg.fac.bacillus.model@formulas[2])), 
    data = elap.gramneg.fac.bacillus, family = binomial)
# Predict outcomes for the data, given the fitted model
predictpr <- predict(fit, type = "response")
# Add the predictions to the dataframe
elap.gramneg.fac.bacillus$PREDICTION <- predictpr
elap.gramneg.fac.bacillus <- data.frame(elap.gramneg.fac.bacillus)
# Create the ROC plot for the best performing model
plot(pROC::roc(elap.gramneg.fac.bacillus$effectiveness ~ elap.gramneg.fac.bacillus$PREDICTION), 
    main = "ROC for best model")
```

```
# Turn probability into a binary response, where > 50%
# probability implies effective venom
elap.gramneg.fac.bacillus <- elap.gramneg.fac.bacillus %>% dplyr::mutate(PREDICTION = ifelse(as.numeric(PREDICTION) < 
    0.5, 0, 1))
# Create the confusion matrix of reality vs prediction
table <- table(Reality = elap.gramneg.fac.bacillus$effectiveness, 
    Prediction = elap.gramneg.fac.bacillus$PREDICTION)
table
```

```
##        Prediction
## Reality  0  1
##       0 13  6
##       1  4 20
```

```
# Calculate accurage for the model
accuracy <- (table[1, 1] + table[2 + 2])/sum(table)
# Display accuracy
paste("The accuracy of this model is: ", accuracy * 100, "%")
```

```
## [1] "The accuracy of this model is:  76.7441860465116 %"
```

```
# Identify false positives from the dataframe
FP <- elap.gramneg.fac.bacillus %>% filter(effectiveness == 0 & 
    PREDICTION == 1) %>% dplyr::select(Snake.species, Phospholipase.A2, 
    Metalloproteinases, Waprin.Kunitz.BPTI)
# Identify false negatives from the dataframe
FN <- elap.gramneg.fac.bacillus %>% filter(effectiveness == 1 & 
    PREDICTION == 0) %>% dplyr::select(Snake.species, Phospholipase.A2, 
    Metalloproteinases, Waprin.Kunitz.BPTI)
```

**False Postives**

| Snake.species | Phospholipase.A2 | Metalloproteinases | Waprin.Kunitz.BPTI |
| --- | --- | --- | --- |
| Naja melanoleuca | 0.4455223 | 0.2796752 | 0.1993068 |
| Naja melanoleuca | 0.4455223 | 0.2796752 | 0.1993068 |
| Naja naja | 0.4058956 | 0.0950112 | 0.0360633 |
| Naja naja | 0.4058956 | 0.0950112 | 0.0360633 |
| Naja naja | 0.4058956 | 0.0950112 | 0.0360633 |
| Naja nigricollis | 0.5066873 | 0.1908939 | 0.0000000 |

**False Negatives**

| Snake.species | Phospholipase.A2 | Metalloproteinases | Waprin.Kunitz.BPTI |
| --- | --- | --- | --- |
| Naja haje | 0.1390113 | 0.306783 | 0.1198695 |
| Naja haje | 0.1390113 | 0.306783 | 0.1198695 |
| Ophiophagus hannah | 0.1854521 | 0.440098 | 0.1471594 |
| Ophiophagus hannah | 0.1854521 | 0.440098 | 0.1471594 |

## AUC Permutation

Having identified Model 2 as the best model, we assessed the ROC curve for sensitivity and specificity. We permuted effectiveness data and recalculated AUC in order to create a distribution of random AUCs. The probability of observing an AUC value greater than or equal to the original AUC value is the p-value.

```
#' This section creates the permutation for AUC values. 
#' Assuming a similar analysis, the user may wish to change: 
#' 1. Number of permutation (currently 1,000)
#' 2. The best fit model (currently model 2)
#' 3. Threshold percentage for defining effective venom (currently 50%)

# Not necessary, but creates starting value for permutation
# index.
i <- 1
# Number of permutations
nreps <- 1000
# Create empty vector for each permuted AUC value
AUC.repo <- rep(0, nreps)
# Copy venom data to preserve integrity
elap.gramneg.fac.bacillus.permute <- elap.gramneg.fac.bacillus
for (i in 1:nreps) {
    # Randomly assign effectiveness value for each venom
    # composition
    elap.gramneg.fac.bacillus.permute$effectiveness <- sample(elap.gramneg.fac.bacillus.permute$effectiveness, 
        size = length(elap.gramneg.fac.bacillus.permute$effectiveness), 
        replace = FALSE)
    # Fit a glm to the randomly sampled data, using the best
    # performing model
    fit <- glm(paste(as.character(elap.gramneg.fac.bacillus.model@formulas[2])), 
        data = elap.gramneg.fac.bacillus, family = binomial)
    # Predict effectiveness given the fitted model
    predictpr <- predict(fit, type = "response")
    # Create an ROC curve for the newly fitted model
    ROC <- pROC::roc(elap.gramneg.fac.bacillus.permute$effectiveness ~ 
        predictpr)
    # Extract the AUC into the empty vector
    AUC.repo[i] <- pROC::auc(ROC)
}
# Create a histogram of AUCs
hist(AUC.repo, xlim = c(0, 1), main = "Histogram of Permuted AUCs")
# Insert a line for the original AUC value
abline(v = elap.gramneg.fac.bacillus.fits$AUC[2], col = "red")
```

```
# Caluclate proportion of AUC values above the original value
prop.above <- length(AUC.repo[AUC.repo > elap.gramneg.fac.bacillus.fits$AUC[1]])/length(AUC.repo)
paste("The p-value for this model is: ", prop.above)
```

```
## [1] "The p-value for this model is:  0"
```

We have no models with a higher AUC, meaning that our model is likely not the product of a random assignment of AUC values.

## Holdout technique cross-validatin

In order to assess whether models may be overfit to our data, we used a holdout procedure, for which we randomly sampled 80% of the data to create a subset to approximate our best performing model. These data are often referred to as “training” data. The remaining 20% of data points were used to assess changes in model accuracy; these data are often referred to as “test” data. In order to assess whether or not our prior accuracies are significantly different from these randomly created training and test sets, we will perform a similar non-parametric procedure as with AUCs. First, we created a randomized training dataset, constituting 80% of the original samples. The best performing model was fit to those data. Predictions and accuracy were assessed from the 20% excluded from the model fitting. This process was repeated 1,000 times, such that we created a distribution of accuracies. We then compared our original accuracy to this distribution. A p-value less than 0.05 suggests that our data were probably not overfit to the original data.

```
#' This section creates the permutation for accuracies. 
#' Assuming a similar analysis, the user may wish to change: 
#' 1. Number of permutation (currently 1,000)
#' 2. Fraction of samples included in the training data (currently 80%)
#' 3. The best fit model (currently model 2)
#' 4. Threshold percentage for defining effective venom (currently 50%)

# Not necessary, but starts index for acc.repo
i <- 1
# Number of permutations
nreps <- 1000
# Creates empty vector of accuracies the length of nreps
acc.repo <- rep(0, nreps)
for (i in 1:nreps) {
    # Creates traning dataset. Here the traning dataset is 80% of
    # the total samples.
    elap.gramneg.fac.bacillus.train <- elap.gramneg.fac.bacillus %>% 
        sample_n(0.8 * nrow(elap.gramneg.fac.bacillus), replace = FALSE)
    
    # Creates the test dataset. Here the test dataset is the
    # other 20% of total samples not included in the training
    # dataset.
    elap.gramneg.fac.bacillus.test <- elap.gramneg.fac.bacillus %>% 
        ## Create a test data set that does not include the training
    ## data
    dplyr::filter(!(row.names(elap.gramneg.fac.bacillus) %in% 
        row.names(elap.gramneg.fac.bacillus.train)))
    
    # Fit a glm using the traning dataset
    fit <- glm(paste(as.character(elap.gramneg.fac.bacillus.model@formulas[2])), 
        data = elap.gramneg.fac.bacillus.train, family = binomial)
    
    # Make predictions using the test dataset
    elap.gramneg.fac.bacillus.test$SUB.PREDICTION <- predict(fit, 
        newdata = elap.gramneg.fac.bacillus.test[, 2:4], type = "response")
    # Mark venoms with a > 50% probability as effective and check
    # whether prediction was correct
    elap.gramneg.fac.bacillus.test <- elap.gramneg.fac.bacillus.test %>% 
        dplyr::mutate(SUB.PREDICTION = ifelse(SUB.PREDICTION < 
            0.5, 0, 1), CORRECT = ifelse(effectiveness == SUB.PREDICTION, 
            1, 0))
    # Calculate accuracy for a given permutation
    acc.repo[i] <- sum(elap.gramneg.fac.bacillus.test$CORRECT)/nrow(elap.gramneg.fac.bacillus.test)
}
# Create a histogram of AUCs
hist(acc.repo, main = "Histogram of Permuted Accurracies")
# Place a line with the calculated accuracy of the original
# model was
abline(v = ((13 + 20)/43), col = "red")
```

```
# Calculate the p-value of that accuracy
p.val <- length(acc.repo[acc.repo > ((13 + 20)/43)])/length(acc.repo)
paste("The p-value for this model's accuracy is", p.val)
```

```
## [1] "The p-value for this model's accuracy is 0.351"
```

Because our p-value is greater than 0.05, we can be confident that our original model was not overfit to our data.

# Implementing new data

In order to implement new data to our models, the following template may be used with little alteration.

```
# Enter filename and TRUE if columns have headers, FALSE if
# absent
new.data <- read.csv("FILENAME.CSV", header = TRUE/FALSE)

# Here model 2 is still selected as the best performing model
# Other models may be selected.
fit <- glm(paste(as.character(elap.gramneg.fac.bacillus.model@formulas[2])), 
    data = elap.gramneg.fac.bacillus, family = binomial)

# New data are entered for prediction Columns will need to be
# selected (Currently XX:XX)
new.data$PREDICTION <- predict(fit, newdata = new.data[, XX:XX], 
    type = "response")

# Display results
new.data
```
