# Supplementary material for: Predicting antibacterial activity from snake venom proteomes
Source: PLoS One. 2020 Jan 24;15(1):e0226807. doi: 10.1371/journal.pone.0226807 (PMC6980403; doi:10.1371/journal.pone.0226807)
Supplement: S2 File — (DOCX) [file pone.0226807.s008.docx]

**Supplemental References**

Abdelkafi-Koubaa Z, Aissa I, Morjen M, Kharrat N, El Ayeb M, Gargouri Y, et al. Interaction of a snake venom L-amino acid oxidase with different cell types membrane. Int J Biol Macromol. 2016;82: 757-764.

Abtahi B, Mosafer Khorjestan S, Ghezellou P, Aliahmadi A, Ranaei Siadat SO, Kazemi SM, et al. Effects of Iranian Snakes Venom True Sea and Terrestrial Snakes on Some Bacterial Cultures. Journal of the Persian Gulf. 2014;5: 27-36.

Accary C, Hraoui-Bloquet S, Hamze M, Mallem Y, El Omar F, Sabatier JM, et al. Protein content analysis and antimicrobial activity of the crude venom of *Montivipera* *bornmuelleri*; a viper from Lebanon. Infect Disord Drug Targets. 2014;14: 49-55.

Aird SD, Aggarwal S, Villar-Briones A, Tin MMY, Terada K, Mikheyev, AS. Snake venoms are integrated systems, but abundant venom proteins evolve more rapidly. BMC genomics. 2015;16: 647.

Alape-Girón A, Sanz L, Escolano J, Flores-Diaz M, Madrigal M, Sasa M, et al. Snake venomics of the lancehead pitviper *Bothrops* *asper*: geographic, individual, and ontogenetic variations. J Proteome Res. 2008;7: 3556-3571.

Al-Asmari AK, Abbasmanthiri R, Osman NMA, Siddiqui Y, Al-Bannah FA, Al-Rawi AM et al. Assessment of the antimicrobial activity of few Saudi Arabian snake venoms. Open Microbiol J. 2015;9: 18.

Angulo Y, Escolano J, Lomonte B, Gutiérrez JM, Sanz L, Calvete JJ, Snake venomics of Central American pitvipers: clues for rationalizing the distinct envenomation profiles of *Atropoides* *nummifer* and Atropoides picadoi. J Proteome Res. 2007;7: 708-719.

Augusto-de-Oliveira C, Stuginski DR, Kitano ES, Andrade-Silva D, Liberato T, Fukushima I, et al. Dynamic rearrangement in snake venom gland proteome: Insights into *Bothrops* *jararaca* intraspecific venom variation. J Proteome Res. 2016;15: 3752-3762.

Barbosa PSF, Martins AMC, Havt A, Toyama DO, Evangelista JSAM, Ferreira DPP, et al. Renal and antibacterial effects induced by myotoxin I and II isolated from *Bothrops* *jararacussu* venom. Toxicon. 2005;46: 376-386.

Barbosa PSF, Martins AMC, Toyama MH, Joazeiro PP, Beriam LOS, Fonteles MC, et al. Purification and biological effects of a C-type lectin isolated from *Bothrops* *moojeni*. J Venom Anim Toxins Incl Trop Dis. 2010;16: 493-504.

Bazaa A, Marrakchi N, El Ayeb M, Sanz L, Calvete JJ. Snake venomics: comparative analysis of the venom proteomes of the Tunisian snakes *Cerastes* *cerastes*, *Cerastes* *vipera* and *Macrovipera* *lebetina*. Proteomics. 2005;5: 4223-4235.

Bernardes CP, Menaldo DL, Camacho E, Rosa JC, Escalante T, Rucavado A, et al. Proteomic analysis of *Bothrops* *pirajai* snake venom and characterization of BpirMP, a new PI metalloproteinase. J Proteomics. 2013;80: 250-267.

Blaylock RS. Antibacterial properties of KwaZulu Natal snake venoms. Toxicon. 2000;38: 1529-1534.

Bocian A, Urbanik M, Hus K, Łyskowski A, Petrilla V, Andrejčáková Z, et al. Proteome and peptidome of *Vipera* *berus* *berus* venom. Molecules. 2016;21: 1398.

Bocian, A, Urbanik M, Hus K, Łyskowski A, Petrilla V, Andrejčáková Z, et al. Proteomic analyses of *Agkistrodon* *contortrix* *contortrix* venom using 2D electrophoresis and MS techniques. Toxins. 2016;8: 372.

Boldrini-França J, Corrêa-Netto C, Silva MM, Rodrigues RS, De La Torre P, Pérez A, et al. Snake venomics and antivenomics of Crotalus durissus subspecies from Brazil: assessment of geographic variation and its implication on snakebite management. J Proteomics. 2010;73: 1758-1776.

Bustillo S, Leiva LC, Merino L, Acosta O, de Kier Joffé EB, Gorodner JO. Antimicrobial activity of *Bothrops* *alternatus* venom from the Northeast of Argentine. Rev Latinoam Microbiol. 2008;50: 79-82.

Calvete JJ. Antivenomics and venom phenotyping: A marriage of convenience to address the performance and range of clinical use of antivenoms. Toxicon. 2010;56: 1284-1291.

Calvete JJ. Snake Venomics, Antivenomics, and Venom Phenotyping: The Ménage à Trois of proteomic tools aimed at understanding the biodiversity of venoms. In: Kini RM, Clemetson K, Markland F, McLane MA, Morita T, editors. Toxins and Hemostasis. Springer; Amsterdam: 2010. pp. 285–300.

Calvete JJ, Borges A, Segura Á, Flores-Díaz M, Alape-Girón A, Gutiérrez JM, et al. Snake venomics and antivenomics of *Bothrops* *colombiensis*, a medically important pitviper of the *Bothrops* *atrox-asper* complex endemic to Venezuela: Contributing to its taxonomy and snakebite management. J Proteomics. 2009;72: 227-240.

Calvete JJ, Escolano J, Sanz L. Snake venomics of *Bitis* species reveals large intragenus venom toxin composition variation: application to taxonomy of congeneric taxa. J Proteome Res. 2007;6: 2732-2745.

Calvete JJ, Fasoli E, Sanz L, Boschetti E, Righetti PG. Exploring the venom proteome of the western diamondback rattlesnake, *Crotalus* *atrox*, via snake venomics and combinatorial peptide ligand library approaches. J Proteome Res. 2009;8: 3055-3067.

Calvete JJ, Ghezellou P, Paiva O, Matainaho T, Ghassempour A, Goudarzi H, et al. Snake venomics of two poorly known Hydrophiinae: Comparative proteomics of the venoms of terrestrial *Toxicocalamus* *longissimus* and marine *Hydrophis* *cyanocinctus*. J Proteomics. 2012;75: 4091-4101.

Calvete JJ, Marcinkiewicz C, Sanz L. Snake venomics of *Bitis* *gabonica* *gabonica*. Protein family composition, subunit organization of venom toxins, and characterization of dimeric disintegrins bitisgabonin-1 and bitisgabonin-2. J Proteome Res. 2006;6: 326-336.

Calvete JJ, Pérez A, Lomonte B, Sánchez EE, Sanz L. Snake venomics of *Crotalus* *tigris*: the minimalist toxin arsenal of the deadliest neartic rattlesnake venom. Evolutionary clues for generating a pan-specific antivenom against crotalid type II venoms. J Proteome Res. 2012;11: 1382-1390.

Calvete JJ, Sanz L, Pérez A, Borges A, Vargas AM, Lomonte B, et al. Snake population venomics and antivenomics of Bothrops atrox: Paedomorphism along its transamazonian dispersal and implications of geographic venom variability on snakebite management. J. Proteomics. 2011;74: 510-527.

Campos PF, Andrade-Silva D, Zelanis A, Paes Leme AF, Rocha MMT, Menezes MC, et al. Trends in the evolution of snake toxins underscored by an integrative omics approach to profile the venom of the colubrid *Phalotris* *mertensi*.Genome Biol Evol. 2016;8: 2266-2287.

Chellapandi P, Jebakumar SRD. Purification and antibacterial activity of Indian cobra and viper venoms. Electron J Biol. 2010;4: 11-16.

Chen LW, Kao PH, Fu YS, Hu WP, Chang LS. Bactericidal effect of *Naja* *nigricollis* toxin γ is related to its membrane-damaging activity. Peptides. 2011;32: 1755-1763.

Chen LW, Kao PH, Fu YS, Lin SR, Chang LS. Membrane-damaging activity of Taiwan cobra cardiotoxin 3 is responsible for its bactericidal activity. Toxicon. 2011;58: 46-53.

Ching AT, Paes Leme AF, Zelanis A, Rocha MM, Furtado MDFD, Silva DA, et al. Venomics profiling of *Thamnodynastes* *strigatus* unveils matrix metalloproteinases and other novel proteins recruited to the toxin arsenal of rear-fanged snakes. J Proteome Res. 2012;11: 1152-1162.

Ciscotto P, De Avila RM, Coelho EAF, Oliveira J, Diniz CG, Farías LM, et al. Antigenic, microbicidal and antiparasitic properties of an L-amino acid oxidase isolated from *Bothrops jararaca* snake venom. Toxicon. 2009; 53: 330-341.

Cisneros Y, Lazo F, Gutiérrez S, Yarlequé A. Biochemical characteristics of an antibacterial protein isolated from the poison of *Lachesis* *muta*. J Chem. Soc. Peru. 2006;72: 1-7.

Conlon JM, Attoub S, Arafat H, Mechkarska M, Casewell NR, Harrison RA, et al. Cytotoxic activities of [Ser49] phospholipase A2 from the venom of the saw-scaled vipers *Echis* *ocellatus*, *Echis* *pyramidum* *leakeyi*, *Echis* *carinatus* *sochureki*, and *Echis* *coloratus*. Toxicon. 201;71: 96-104.

Corrêa EA, Kayano AM, Diniz-Sousa R, Setúbal SS, Zanchi FB, Zuliani JP, et al. Isolation, structural and functional characterization of a new Lys49 phospholipase A2 homologue from *Bothrops* *neuwiedi* *urutu* with bactericidal potential. Toxicon. 2016;115: 13-21.

Corrêa-Netto C, de LM Junqueira-de-Azevedo I, Silva DA, Ho PL, Leitão-de-Araújo M, Alves M, et al. Snake venomics and venom gland transcriptomic analysis of Brazilian coral snakes, *Micrurus* *altirostris* and *M*. *corallinus*. J Proteomics. 2011;74: 1795-1809.

Costa TR, Menaldo DL, da Silva CP, Sorrechia R, de Albuquerque S, Pietro RC, et al. Evaluating the microbicidal, antiparasitic and antitumor effects of CR-LAAO from *Calloselasma rhodostoma* venom. Int J Biol Macromol. 2015;80: 489-497.

Costa TR, Menaldo DL, Oliveira CZ, Santos-Filho NA, Teixeira SS, Nomizo A, et al. Myotoxic phospholipases A2 isolated from *Bothrops* *brazili* snake venom and synthetic peptides derived from their C-terminal region: cytotoxic effect on microorganism and tumor cells. Peptides. 2008;29: 1645-1656.

dos Santos Nunes E, De Souza MAA, de Melo Vaz AF, de Sá Santana GM, Gomes FS, Coelho LCBB, et Purification of a lectin with antibacterial activity from *Bothrops* *leucurus* snake venom. Comp Biochem Physiol B Biochem Mol Biol. 2011;159: pp.57-63.

Durban J, Pérez A, Sanz L, Gómez A, Bonilla F, Rodríguez S, et al. Integrated “omics” profiling indicates that miRNAs are modulators of the ontogenetic venom composition shift in the Central American rattlesnake, *Crotalus* *simus* *simus*. BMC genomics. 2013;14: 234.

Dutta S, Chanda A, Kalita B, Islam T, Patra A, Mukherjee AK. Proteomic analysis to unravel the complex venom proteome of eastern India *Naja* *naja*: Correlation of venom composition with its biochemical and pharmacological properties. J Proteomics. 2017;156: 29-39.

Fahmi L, Makran B, Pla D, Sanz L, Oukkache N, Lkhider M, et al. Venomics and antivenomics profiles of North African *Cerastes* *cerastes* and *C*. *vipera* populations reveals a potentially important therapeutic weakness. J Proteomics. 2012;75: 2442-2453.

Fathi B, Jamshidi A, Zolfagharian H, Zare Mirakabbadi A. Investigation of the antibacterial effect of venom of the Iranian snake *Echis* *carinatus*. Iran J Vet Sci Technol. 2011;2: 93-99.

Fernández J, Alape-Girón A, Angulo Y, Sanz L, Gutiérrez JM, Calvete JJ, et al. Venomic and antivenomic analyses of the Central American coral snake, *Micrurus* *nigrocinctus* (Elapidae). J Proteome Res. 2011;10: 1816-1827.

Fernández J, Lomonte B, Sanz L, Angulo Y, Gutiérrez JM, Calvete JJ. Snake venomics of *Bothriechis* *nigroviridis* reveals extreme variability among palm pitviper venoms: different evolutionary solutions for the same trophic purpose. J Proteome Res. 201;9: 4234-4241.

Fernández J, Vargas-Vargas N, Pla D, Sasa M, Rey-Suárez P, Sanz L, et al. Snake venomics of *Micrurus* *alleni* and *Micrurus* *mosquitensis* from the Caribbean region of Costa Rica reveals two divergent compositional patterns in New World elapids. Toxicon. 2015;107: 217-233.

Ferreira BL, Santos DO, Santos ALD, Rodrigues CR, de Freitas CC, Cabral LM, et al. Comparative analysis of viperidae venoms antibacterial profile: a short communication for proteomics. Evid Based Complement Alternat Med. 2011;2011: 960267.

Florea ŞAG, Ciurca D, Mare A, Man A, Cordoş B, Grama AL, Muntean DL. New RP-HPLC Method for Separation of *Naja* *haje* *haje* Venom and Studies of its Bactericidal Effect. Acta Med Marisiensis. 2016;62: 90-94.

Francischetti IM, My-Pham V, Harrison J, Garfield MK, Ribeiro JM. Bitis gabonica (Gaboon viper) snake venom gland: toward a catalog for the full-length transcripts (cDNA) and proteins. Gene. 2004;337: 55-69

Gay C, Sanz L, Calvete JJ, Pla D. Snake venomics and antivenomics of *Bothrops* *diporus*, a medically important pitviper in northeastern Argentina. Toxins. 2016;8: 9.

Georgieva D, Ohler M, Seifert J, Bergen MV, Arni RK, Genov N, et al. Snake Venomic of *Crotalus* *durissus* *terrificus* Correlation with Pharmacological Activities. J Proteome Res. 2010;9: 2302-2316.

Georgieva D, Seifert J, Ohler M, von Bergen M, Spencer P, Arni RK, et al. *Pseudechis* *australis* venomics: adaptation for a defense against microbial pathogens and recruitment of body transferrin. J Proteome Res. 2011;10: 2440-2464.

Gibbs HL, Sanz L, Sovic MG, Calvete JJ. Phylogeny-based comparative analysis of venom proteome variation in a clade of rattlesnakes (*Sistrurus* sp.). PloS One. 2013;8: e67220.

Glaser HR. Bactericidal activity of *Crotalus* venom in vitro. Copeia. 1948;1948: 245-247.

Göçmen B, Heiss P, Petras D, Nalbantsoy A, Süssmuth RD. Mass spectrometry guided venom profiling and bioactivity screening of the Anatolian Meadow Viper, *Vipera* *anatolica*. Toxicon. 2015;107: 163-174.

Gonçalves-Machado L, Pla D, Sanz L, Jorge RJB, Leitão-De-Araújo M, Alves MLM, et al. Combined venomics, venom gland transcriptomics, bioactivities, and antivenomics of two *Bothrops* *jararaca* populations from geographic isolated regions within the Brazilian Atlantic rainforest. J Proteomics. 2016;135: 73-89.

Gutiérrez JM, Sanz L, Escolano J, Fernández J, Lomonte B, Angulo Y, et al. Snake venomics of the Lesser Antillean pit vipers Bothrops caribbaeus and Bothrops lanceolatus: correlation with toxicological activities and immunoreactivity of a heterologous antivenom. J Proteome Res. 2008;7: 4396-4408.

Hakim MA, Reza MA. In vitro antibacterial activity of snake venom, *Naja* *naja* from Bangladesh. Br Biotechnol J. 2015;8: 1-5

Hanane‐Fadila ZM, Fatima LD. Purification, Characterization and Antibacterial Activity of l‐amino Acid Oxidase from Cerastes cerastes. J Biochem Mol Tox. 2014;28: 347-354.

Igci N, Demiralp DO. A preliminary investigation into the venom proteome of *Macrovipera* *lebetina* *obtusa* (Dwigubsky, 1832) from Southeastern Anatolia by MALDI-TOF mass spectrometry and comparison of venom protein profiles with *Macrovipera* *lebetina* *lebetina* (Linnaeus, 1758) from Cyprus by 2D-PAGE. Arch Toxicol. 2012;86: 441-451.

Iglesias CV, Aparicio R, Rodrigues-Simioni L, Camargo EA, Antunes E, Marangoni S, et al. Effects of morin on snake venom phospholipase A2 (PLA2). Toxicon. 2005;46: 751-758.

Izidoro LFM, Ribeiro MC, Souza GR, Sant’Ana CD, Hamaguchi A, Homsi-Brandeburgo MI, et al. Biochemical and functional characterization of an L-amino acid oxidase isolated from *Bothrops* *pirajai* snake venom. Bioorg Med Chem. 2006;14: 7034-7043.

Jiménez-Charris E, Montealegre-Sanchez L, Solano-Redondo L, Mora-Obando D, Camacho E, Castro-Herrera F, et al. Proteomic and functional analyses of the venom of *Porthidium* *lansbergii* *lansbergii* (Lansberg's hognose viper) from the Atlantic Department of Colombia. J Proteomics. 2015;114: 287-299.

Jorge RJB, Monteiro HS, Gonçalves-Machado L, Guarnieri MC, Ximenes RM, Borges-Nojosa DM, et al. Venomics and antivenomics of *Bothrops* *erythromelas* from five geographic populations within the Caatinga ecoregion of northeastern Brazil. J Proteomics. 2015;114: 93-114.

Kalayci S, Ustaoglu Iyigundogdu Z, Yazici M, Asutay B, Demir O, Sahin F. Evaluation of antimicrobial and antiviral activities of different venoms. Infect Disord Drug Targets. 2016;16: 44-53.

Kalita B, Patra A, Mukherjee AK. Unraveling the proteome composition and immuno-profiling of western India Russell’s viper venom for in-depth understanding of its pharmacological properties, clinical manifestations, and effective antivenom treatment. J Proteome Res. 2016;16: 583-598.

Klein RC, Fabres-Klein MH, de Oliveira LL, Feio RN, Malouin F, Ribon ADOB. A C-type lectin from *Bothrops* *jararacussu* venom disrupts Staphylococcal biofilms. PLoS One. 2015;10: e0120514.

Kohlhoff M, Borges MH, Yarleque A, Cabezas C, Richardson M, Sanchez EF. Exploring the proteomes of the venoms of the Peruvian pit vipers *Bothrops* *atrox*, *B*. *barnetti* and *B*. *pictus*. J Proteomics. 2012;75: 2181-2195.

Kovalchuk S, Ziganshin R, Starkov V, Tsetlin V, Utkin Y. Quantitative proteomic analysis of venoms from Russian vipers of *Pelias* group: Phospholipases A2 are the main venom components. Toxins. 2016;8: 105.

Latinović Z, Leonardi A, Šribar J, Sajevic T, Žužek MC, Frangež R, et al. Venomics of *Vipera* *berus* *berus* to explain differences in pathology elicited by *Vipera* ammodytes ammodytes envenomation: Therapeutic implications. J Proteomics. 2016;146: 34-47.

Lauridsen LP, Laustsen AH, Lomonte B, Gutiérrez JM. Toxicovenomics and antivenom profiling of the Eastern green mamba snake (*Dendroaspis* *angusticeps*). J Proteomics. 2016;136: 248-261.

Lauridsen LP, Laustsen AH, Lomonte B, Gutiérrez JM. Exploring the venom of the forest cobra snake: Toxicovenomics and antivenom profiling of *Naja* *melanoleuca*. J Proteomics. 2017;150: 98-108.

Lazo F, Málaga O, Yarlequé A, Severino R, Gutiérrez S. Actividad antimicrobiana de una flavoproteína aislada del veneno de la serpiente peruana *Bothrops* *atrox* (" jergón"). Revista de la Sociedad Química del Perú. 2007;73: 197-207.

Lee ML, Tan NH, Fung SY, Sekaran SD. Antibacterial action of a heat-stable form of L-amino acid oxidase isolated from king cobra (*Ophiophagus* *hannah*) venom. Comp Biochem Physiol C Toxicol Pharmacol. 2011;153: 237-242.

Liu JW, Chai MQ, Du XY, Song JG, Zhou YC.Purification and characterization of L-amino acid oxidase from *Agkistrodon* *halys* *pallas* venom. Acta biochimica et biophysica Sinica. 2002;34: 305-310.

Lomonte B, Escolano J, Fernández J, Sanz L, Angulo Y, Gutiérrez JM, et al. Snake venomics and antivenomics of the arboreal neotropical pitvipers *Bothriechis* *lateralis* and *Bothriechis* *schlegelii*. J Proteome Res. 2008;7: 2445-2457.

Lomonte B, Fernández J, Sanz L, Angulo Y, Sasa M, Gutiérrez JM, et al. Venomous snakes of Costa Rica: Biological and medical implications of their venom proteomic profiles analyzed through the strategy of snake venomics. J Proteomics. 2014;105: 323-339.

Lomonte B, Pla D, Sasa M, Tsai WC, Solórzano A, Ureña-Díaz JM, et al. Two color morphs of the pelagic yellow-bellied sea snake, *Pelamis* *platura*, from different locations of Costa Rica: snake venomics, toxicity, and neutralization by antivenom. J Proteomics. 2014;103: 137-152.

Lomonte B, Rey-Suárez P, Fernández J, Sasa M, Pla D, Vargas N, et al. Venoms of *Micrurus* coral snakes: Evolutionary trends in compositional patterns emerging from proteomic analyses. Toxicon. 2016;122: 7-25.

Lomonte B, Rey-Suárez P, Tsai WC, Angulo Y, Sasa M, Gutiérrez JM, et al. Snake venomics of the pit vipers *Porthidium* *nasutum*, *Porthidium* *ophryomegas*, and *Cerrophidion* *godmani* from Costa Rica: toxicological and taxonomical insights. J Proteomics. 2012;75: 1675-1689.

Lomonte B, Tsai WC, Bonilla F, Solórzano A, Solano G, Angulo Y, et al. Snake venomics and toxicological profiling of the arboreal pitviper *Bothriechis* *supraciliaris* from Costa Rica. Toxicon. 2012;59: 592-599.

Lomonte B, Tsai WC, Ureña-Diaz JM, Sanz L, Mora-Obando D, Sánchez EE, et al. Venomics of New World pit vipers: genus-wide comparisons of venom proteomes across *Agkistrodon*. J Proteomics. 2014;96: 103-116.

Lu QM, Wei Q, Jin Y, Wei JF, Wang WY, Xiong YL. L-amino acid oxidase from *Trimeresurus* *jerdonii* snake venom: purification, characterization, platelet aggregation-inducing and antibacterial effects. J Nat Toxins. 2002;11: 345-352.

Madrigal M, Sanz L, Flores-Díaz M, Sasa M, Núñez V, Alape-Girón A, et al. Snake venomics across genus Lachesis. Ontogenetic changes in the venom composition of *Lachesis* *stenophrys* and comparative proteomics of the venoms of adult *Lachesis* *melanocephala* and *Lachesis* *acrochorda*. J Proteomics. 2012;77: 280-297.

Makran B, Fahmi L, Pla D, Sanz L, Oukkache N, Lkhider M, et al. Snake venomics of *Macrovipera* *mauritanica* from Morocco, and assessment of the para-specific immunoreactivity of an experimental monospecific and a commercial antivenoms. J Proteomics. 2012;75: 2431-2441.

Mora-Obando D, Guerrero-Vargas JA, Prieto-Sánchez R, Beltrán J, Rucavado A, Sasa M, et al. Proteomic and functional profiling of the venom of *Bothrops* *ayerbei* from Cauca, Colombia, reveals striking interspecific variation with *Bothrops* *asper* venom. J Proteomics. 2014;96: 159-172.

Mukherjee AK, Kalita B, Mackessy SP. A proteomic analysis of Pakistan *Daboia* *russelii* *russelii* venom and assessment of potency of Indian polyvalent and monovalent antivenom. J Proteomics. 2016;144: 73-86.

Mora JM. Caracterización proteómica de venenos de serpientes de interés biomédico. Ph.D. Dissertation, Universitat Politècnica de València. 2016. Available from https://riunet.upv.es/bitstream/handle/10251/70417/MUNUERA%20-%20Caracterizaci%C3%B3n%20prote%C3%B3mica%20de%20venenos%20de%20serpientes%20de%20inter%C3%A9s%20biom%C3%A9dico.pdf?sequence=1

Nair DG, Fry BG, Alewood P, Kumar PP, Kini RM. Antimicrobial activity of omwaprin, a new member of the waprin family of snake venom proteins. Biochem J. 2007;402: 93-104.

Núñez V, Cid P, Sanz L, De La Torre P, Angulo Y, Lomonte B, et al. Snake venomics and antivenomics of *Bothrops* *atrox* venoms from Colombia and the Amazon regions of Brazil, Perú and Ecuador suggest the occurrence of geographic variation of venom phenotype by a trend towards paedomorphism. J Proteomics. 2009;73: 57-78.

Oguiura N, Boni-Mitake M, Affonso R, Zhang G. In vitro antibacterial and hemolytic activities of crotamine, a small basic myotoxin from rattlesnake *Crotalus* *durissus*. J Antibiot. 2011;64: 327.

Öhler M, Georgieva D, Seifert J, von Bergen M, Arni RK, Genov N, et al. The venomics of *Bothrops* *alternatus* is a pool of acidic proteins with predominant hemorrhagic and coagulopathic activities. J Proteome Res. 2010;9: 2422-2437.

Okubo BM, Silva ON, Migliolo L, Gomes DG, Porto WF, Batista CL, et al. Evaluation of an antimicrobial L-amino acid oxidase and peptide derivatives from *Bothropoides* *mattogrossense* pitviper venom. PloS One. 2012;7: e33639.

Pahari S, Mackessy SP, Kini RM. The venom gland transcriptome of the Desert Massasauga Rattlesnake (*Sistrurus* *catenatus* *edwardsii*): towards an understanding of venom composition among advanced snakes (Superfamily Colubroidea). BMC Mol Biol. 2007;8: 115.

Paiva O, Pla D, Wright CE, Beutler M, Sanz L, Gutiérrez JM, et al. Combined venom gland cDNA sequencing and venomics of the New Guinea small-eyed snake, *Micropechis* *ikaheka*. J Proteomics. 2014;110: 209-229.

Paiva RDMA, de Freitas Figueiredo R, Antonucci GA, Paiva HH, Bianchi MDLP, Rodrigues KC, et al. Cell cycle arrest evidence, parasiticidal and bactericidal properties induced by L-amino acid oxidase from *Bothrops* *atrox* snake venom. Biochimie. 2011;93: 941-947.

Páramo L, Lomonte B, Pizarro‐Cerdá J, Bengoechea JA, Gorvel JP, Moreno E. Bactericidal activity of Lys49 and Asp49 myotoxic phospholipases A2 from *Bothrops* *asper* snake venom: Synthetic Lys49 myotoxin II‐(115− 129)‐peptide identifies its bactericidal region. Eur J Biochem. 1998;253: 452-461.

Petras D, Heiss P, Süssmuth RD, Calvete JJ. Venom proteomics of Indonesian king cobra, *Ophiophagus* *hannah*: integrating top-down and bottom-up approaches. J Proteome Res. 2015;14: 2539-2556.

Phua CS, Vejayan J, Ambu S, Ponnudurai G, Gorajana A. Purification and antibacterial activities of an L-amino acid oxidase from king cobra (*Ophiophagus* *hannah*) venom. J Venom Anim Toxins Incl Trop Dis. 2012;18: 198-207.

Pla D, Bande BW, Welton RE, Paiva OK, Sanz L, Segura A, et al. Proteomics and antivenomics of Papuan black snake (*Pseudechis* *papuanus*) venom with analysis of its toxicological profile and the preclinical efficacy of Australian antivenoms. J Proteomics. 2017;150: 201-215.

Pla D, Sanz L, Molina-Sánchez P, Zorita V, Madrigal M, Flores-Díaz M, et al. Snake venomics of *Lachesis* *muta* *rhombeata* and genus-wide antivenomics assessment of the paraspecific immunoreactivity of two antivenoms evidence the high compositional and immunological conservation across *Lachesis*. J Proteomics. 2013;89: 112-123.

Pla D, Sanz L, Sasa M, Acevedo ME, Dwyer Q, Durban J, et al. Proteomic analysis of venom variability and ontogeny across the arboreal palm-pitvipers (genus *Bothriechis*). J Proteomics. 2017;152: 1-12.

Pla D, Sanz L, Whiteley G, Wagstaff SC, Harrison RA, Casewell NR, et al. What killed Karl Patterson Schmidt? Combined venom gland transcriptomic, venomic and antivenomic analysis of the South African green tree snake (the boomslang), *Dispholidus* *typus*. Biochim Biophys Acta (BBA)-General Subjects. 2017;1861: 814-823.

Prabhakaran AK, Kumaravel P, Priya J, Melchias G, Edward A, Sridevi G. Investigation of antibacterial and haemolytic activity of Russell’s viper and *Echis* *carinatus* venom. Asian J Pharm Anal. 2014;4: 1-4.

Rádis-Baptista G, Moreno FBMB, de Lima Nogueira L, Martins AM, de Oliveira Toyama D, Toyama MH, et al. Crotacetin, a novel snake venom C-type lectin homolog of convulxin, exhibits an unpredictable antimicrobial activity. Cell Biochem Biophys. 2006;44: 412-423.

Resende LM, Almeida JR, Schezaro-Ramos R, Collaço RCO, Simioni LR, Ramirez D, et al. Exploring and understanding the functional role, and biochemical and structural characteristics of an acidic phospholipase A2, AplTx-I, purified from *Agkistrodon* *piscivorus* *leucostoma* snake venom. Toxicon. 2017;127: 22-36.

Rey-Suárez P, Núñez V, Fernández J, Lomonte B. Integrative characterization of the venom of the coral snake *Micrurus* *dumerilii* (Elapidae) from Colombia: Proteome, toxicity, and cross-neutralization by antivenom. J Proteomics. 2016;136: 262-273.

Rey-Suárez P, Núñez V, Gutiérrez JM, Lomonte B. Proteomic and biological characterization of the venom of the redtail coral snake, *Micrurus* *mipartitus* (Elapidae), from Colombia and Costa Rica. J Proteomics. 2011;75: 655-667.

Rima M, Accary C, Haddad K, Sadek R, Hraoui-Bloquet S, Desfontis J, et al. Identification of L-amino acid oxidase (Mb-LAAO) with antibacterial activity in the venom of *Montivipera* *bornmuelleri*, a viper from Lebanon. Infect Disord Drug Targets. 2013;13: 337-343.

Roberto PG, Kashima S, Marcussi S, Pereira JO, Astolfi-Filho S, Nomizo A, et al. Cloning and identification of a complete cDNA coding for a bactericidal and antitumoral acidic phospholipase A 2 from *Bothrops* *jararacussu* venom. Protein J. 2004;23: 273-285.

Rodrigues RS, Boldrini-França J, Fonseca FP, de la Torre P, Henrique-Silva F, Sanz L, et al. Combined snake venomics and venom gland transcriptomic analysis of *Bothropoides* *pauloensis*. J Proteomics. 2012;75: 2707-2720.

Rodrigues VM, Marcussi S, Cambraia RS, de Araújo AL, Malta-Neto NR, Hamaguchi A, et al. Bactericidal and neurotoxic activities of two myotoxic phospholipases A2 from *Bothrops* *neuwiedi* *pauloensis* snake venom. Toxicon. 2004;44: 305-314.

Sachidananda MK, Murari SK, Channe Gowda D. Characterization of an antibacterial peptide from Indian cobra (*Naja* *naja*) venom. J Venom Anim Toxins Incl Trop Dis. 2007;13: 446-461.

Salazar-Valenzuela D, Mora-Obando D, Fernández ML, Loaiza-Lange A, Gibbs HL, Lomonte B. Proteomic and toxicological profiling of the venom of *Bothrocophias* *campbelli*, a pitviper species from Ecuador and Colombia. Toxicon. 2014;90: 15-25.

Samel M, Tõnismägi K, Rönnholm G, Vija H, Siigur J, Kalkkinen N, et al. L-Amino acid oxidase from *Naja* *naja* *oxiana* venom. Comp Biochem Physiol B Biochem Mol Biol. 2008;149: 572-580.

Samel M, Vija H, Kurvet I, Künnis-Beres K, Trummal K, Subbi J, et al. Interactions of PLA2-s from *Vipera* *lebetina*, *Vipera* *berus* *berus* and *Naja* *naja* *oxiana* venom with platelets, bacterial and cancer cells. Toxins. 2013;5: 203-223.

Samy RP, Gopalakrishnakone P, Bow H, Puspharaj PN, Chow, VT. Identification and characterization of a phospholipase A2 from the venom of the Saw-scaled viper: Novel bactericidal and membrane damaging activities. Biochimie. 2010;92: 1854-1866.

Samy RP, Gopalakrishnakone P, Chow VT, Ho B. Viper metalloproteinase (*Agkistrodon* *halys* *pallas*) with antimicrobial activity against multi‐drug resistant human pathogens. J Cell Phys. 2008;216: 54-68.

Samy RP, Gopalakrishnakone P, Ho B, Chow VT. Purification, characterization and bactericidal activities of basic phospholipase A2 from the venom of *Agkistrodon* *halys* (Chinese *pallas*). Biochimie. 2008;90: 1372-1388.

Samy RP, Kandasamy M, Gopalakrishnakone P, Stiles BG, Rowan EG, Becker D, et al. Wound healing activity and mechanisms of action of an antibacterial protein from the venom of the eastern diamondback rattlesnake (*Crotalus* *adamanteus*). PLoS One. 2014;9: e80199.

Samy RP, Pachiappan A, Gopalakrishnakone P, Thwin MM, Hian YE, Chow VT, et al. In vitro antimicrobial activity of natural toxins and animal venoms tested against *Burkholderia* *pseudomallei*. BMC Infect Dis. 2006;6: 100.

Samy RP, Stiles BG, Chinnathambi A, Zayed ME, Alharbi SA, Franco OL, et al. Viperatoxin‐II: A novel viper venom protein as an effective bactericidal agent. FEBS Open Bio. 2015;5: 928-941.

San TM, Vejayan J, Shanmugan K, Ibrahim H. Screening antimicrobial activity of venoms from snakes commonly found in Malaysia. J Appl Sci. (Faisalabad). 2010;10: 2328-2332.

Santamaría C, Larios S, Angulo Y, Pizarro-Cerda J, Gorvel JP, Moreno E, et al. Antimicrobial activity of myotoxic phospholipases A2 from crotalid snake venoms and synthetic peptide variants derived from their C-terminal region. Toxicon. 2005;45: 807-815.

Sanz L, Ayvazyan N, Calvete JJ. Snake venomics of the Armenian mountain vipers *Macrovipera* *lebetina* *obtusa* and *Vipera* *raddei*. J Proteomics. 2008;71: 198-209.

Sanz L, Escolano J, Ferretti M, Biscoglio MJ, Rivera E, Crescenti EJ, et al. Snake venomics of the South and Central American Bushmasters. Comparison of the toxin composition of *Lachesis* *muta* gathered from proteomic versus transcriptomic analysis. J Proteomics. 2008;71: 46-60.

Sanz L, Gibbs HL, Mackessy SP, Calvete JJ. Venom proteomes of closely related *Sistrurus* rattlesnakes with divergent diets. J Proteome Res. 2006;5: 2098-2112.

Sanz L, Pla D, Pérez A, Rodríguez Y, Zavaleta A, Salas M, et al. Venomic analysis of the poorly studied desert coral snake, *Micrurus* *tschudii* *tschudii*, supports the 3FTx/PLA2 dichotomy across *Micrurus* venoms. Toxins. 2016;8: 178.

Saviola AJ, Pla D, Sanz L, Castoe TA, Calvete JJ, Mackessy SP. Comparative venomics of the Prairie Rattlesnake (*Crotalus* *viridis* *viridis*) from Colorado: Identification of a novel pattern of ontogenetic changes in venom composition and assessment of the immunoreactivity of the commercial antivenom CroFab®. J Proteomics. 2015;121: 28-43.

Shan LL, Gao JF, Zhang YX, Shen SS, He Y, Wang J, et al. Proteomic characterization and comparison of venoms from two elapid snakes (*Bungarus* *multicinctus* and *Naja* *atra*) from China. J Proteomics. 2016;138: 83-94.

Shebl RI, Mohamed AF, Ali AE, Amin MA. Antimicrobial profile of selected snake venoms and their associated enzymatic activities. Br Microbiol Res J. 2012;2: 251-263.

Sintiprungrat K, Watcharatanyatip K, Senevirathne WDST, Chaisuriya P, Chokchaichamnankit D, Srisomsap C, et al. A comparative study of venomics of *Naja* *naja* from India and Sri Lanka, clinical manifestations and antivenomics of an Indian polyspecific antivenom. J Proteomics. 2016;132: 131-143.

Solís C, Escobar E, Yarlequé A, Gutiérrez S. Purificación y caracterización de la l-amino ácido oxidasa del veneno de la serpiente *Bothrops* *brazili*" jergón shushupe". Rev Peru Biol. 1999;6: 075-084.

Stábeli RG, Amui SF, Sant'Ana CD, Pires MG, Nomizo A, Monteiro MC, et al. *Bothrops* *moojeni* myotoxin-II, a Lys49-phospholipase A2 homologue: an example of function versatility of snake venom proteins. Comp Biochem Physiol C Toxicol Pharmacol. 2006;142: 371-381.

Stábeli RG, Marcussi S, Carlos GB, Pietro RC, Selistre-de-Araújo HS, Giglio JR, et al. Platelet aggregation and antibacterial effects of an L-amino acid oxidase purified from *Bothrops* *alternatus* snake venom. Bioorg Med Chem. 2004;12: 2881-2886.

Stiles BG, Sexton FW, Weinstein SA. Antibacterial effects of different snake venoms: purification and characterization of antibacterial proteins from *Pseudechis* *australis* (Australian king brown or mulga snake) venom. Toxicon. 1991;29: 1129-1141.

Stocker JF, Traynor JR. The action of various venoms on *Escherichia* *coli*. J Appl Bacteriol. 1986;61: 383-388.

Sudarshan S, Dhananjaya BL. Antibacterial potential of a basic phospholipase A 2 (VRV-PL-V) of *Daboia* *russellii* *pulchella* (Russell’s viper) venom. Biochem. (Moscow). 2014;79: 1237-1244.

Sudarshan S, Dhananjaya BL. The antimicrobial activity of an acidic phospholipase A 2 (NN-XIa-PLA 2) from the venom of *Naja* *naja* *naja* (Indian Cobra). Appl Biochem Biotechnol. 2015;176: 2027-2038.

Sulca MA, Remuzgo C, Cárdenas J, Kiyota S, Cheng E, Bemquerer MP, et al. Venom of the Peruvian snake *Bothriopsis* *oligolepis*: Detection of antibacterial activity and involvement of proteolytic enzymes and C-type lectins in growth inhibition of *Staphylococcus* *aureus*. Toxicon. 2017;134: 30-40.

Sun MZ, Guo C, Tian Y, Chen D, Greenaway FT, Liu S. Biochemical, functional and structural characterization of Akbu-LAAO: a novel snake venom L-amino acid oxidase from *Agkistrodon* *blomhoffii* *ussuriensis*. Biochimie. 2010;92: 343-349.

Talan DA, Citron DM, Overturf GD, Singer B, Froman P, Goldstein EJ. Antibacterial activity of crotalid venoms against oral snake flora and other clinical bacteria. J Infect Dis. 1991;164: 195-198.

Talebi MM, Madani R, Hajihosseini R, Moradi SB. Antibacterial activity of isolated immunodominant proteins of *Naja* *naja* (*oxiana*) venom. Iran J Phar Res. 2017;16: 297-305.

Tan CH, Fung SY, Yap MKK, Leong PK, Liew JL, Tan NH. Unveiling the elusive and exotic: Venomics of the Malayan blue coral snake (*Calliophis* *bivirgata* *flaviceps*). J Proteomics. 2016;132: 1-12.

Tan CH, Tan KY, Fung SY, Tan NH. Venom-gland transcriptome and venom proteome of the Malaysian king cobra (*Ophiophagus* *hannah*). BMC Genomics. 2015;16: 687.

Tan CH, Tan KY, Lim SE, Tan NH. Venomics of the beaked sea snake, *Hydrophis* *schistosus*: A minimalist toxin arsenal and its cross-neutralization by heterologous antivenoms. J Proteomics. 2015;126: 121-130.

Tan CH, Tan KY, Yap MKK, Tan NH. Venomics of *Tropidolaemus* *wagleri*, the sexually dimorphic temple pit viper: Unveiling a deeply conserved atypical toxin arsenal. Sci Rep. 2017;7: 43237.

Tan CH, Tan KY, Tan NH. Revisiting *Notechis* *scutatus* venom: on shotgun proteomics and neutralization by the “bivalent” sea snake antivenom. J Proteomics. 2016;144: 33-38.

Tan CH, Tan NH, Sim SM, Fung SY, Gnanathasan CA. Proteomic investigation of Sri Lankan hump-nosed pit viper (*Hypnale* *hypnale*) venom. Toxicon. 2015;93: 164-170.

Tan NH, Wong KY, Tan CH. Venomics of *Naja* *sputatrix*, the Javan spitting cobra: A short neurotoxin-driven venom needing improved antivenom neutralization. J Proteomics. 2017;157: 18-32.

Tang ELH, Tan CH, Fung SY, Tan NH. Venomics of *Calloselasma* *rhodostoma*, the Malayan pit viper: A complex toxin arsenal unraveled. J Proteomics. 2016;148: 44-56.

Tashima AK, Sanz L, Camargo AC, Serrano SM, Calvete JJ. Snake venomics of the Brazilian pitvipers *Bothrops* *cotiara* and *Bothrops* *fonsecai*. Identification of taxonomy markers. J Proteomics. 2008;71: 473-485.

Tõnismägi K, Samel M, Trummal K, Rönnholm G, Siigur J, Kalkkinen N, et al. L-amino acid oxidase from *Vipera* *lebetina* venom: isolation, characterization, effects on platelets and bacteria. Toxicon. 2006;48: 227-237.

Torres AFC, Dantas RT, Toyama MH, Diz Filho E, Zara FJ, de Queiroz MGR, et al. Antibacterial and antiparasitic effects of *Bothrops* *marajoensis* venom and its fractions: phospholipase A2 and L-amino acid oxidase. Toxicon. 2010;55: 795-804.

Toyama MH, de Oliveira DG, Beriam LO, Novello JC, Rodrigues-Simioni L, Marangoni S. Structural, enzymatic and biological properties of new PLA2 isoform from *Crotalus* *durissus* *terrificus* venom. Toxicon. 2003;41: 1033-1038.

Toyama MH, Toyama DDO, Passero LF, Laurenti MD, Corbett CE, Tomokane TY, et al. Isolation of a new L-amino acid oxidase from *Crotalus* *durissus* *cascavella* venom. Toxicon. 2006;47: 47-57.

Valente RH, Guimarães PR, Junqueira M, Neves-Ferreira AGC, Soares MR, Chapeaurouge A, et al. *Bothrops insularis* venomics: a proteomic analysis supported by transcriptomic-generated sequence data. J Proteomics. 2009;72: 241-255.

Vargas LJ, Londoño M, Quintana JC, Rua C, Segura C, Lomonte B, et al. An acidic phospholipase A2 with antibacterial activity from *Porthidium nasutum* snake venom. Comp Biochem Physiol B Biochem Mol Biol. 2012;161: 341-347.

Vargas Munoz LJ, Quintana JC, Pereañez JA, Núñez V, Sanz L, Calvete JJ. Cloning and characterization of an antibacterial L-amino acid oxidase from *Crotalus durissus cumanensis* venom. Toxicon. 2013;64: 1-11.

Vargas Munoz LJ, Estrada-Gomez S, Nunez V, Sanz L, Calvete JJ. Characterization and cDNA sequence of *Bothriechis schlegelii* amino acid oxidase with antibacterial activity. Int J Biol Macromol. 2014;69: 200-7.

Villalta M, Pla D, Yang SL, Sanz L, Segura A, Vargas M, et al. Snake venomics and antivenomics of *Protobothrops mucrosquamatus* and *Viridovipera stejnegeri* from Taiwan: keys to understand the variable immune response in horses. J Proteomics. 2012;75: 5628-5645.

Wagstaff SC, Sanz L, Juárez P, Harrison RA, Calvete JJ. Combined snake venomics and venom gland transcriptomic analysis of the ocellated carpet viper, *Echis ocellatus*. J Proteomics. 2009;71: 609-623.

Wang Y, Hong J, Liu X, Yang H, Liu R, Wu J, et al. Snake cathelicidin from *Bungarus fasciatus* is a potent peptide antibiotics. PloS One. 2008;3: e3217.

Xie JP, Yue J, Xiong YL, Wang WY, Yu SQ, Wang HH. In vitro activities of small peptides from snake venom against clinical isolates of drug-resistant *Mycobacterium tuberculosis*. Int J Antimicrob Agents. 2003;22: 172-174.

Xu C, Ma D, Yu H, Li Z, Liang J, Lin G, et al. A bactericidal homodimeric phospholipases A2 from *Bungarus fasciatus* venom. Peptides. 2007;28: 969-973.

Xu N, Zhao HY, Yin Y, Shen SS, Shan LL, Chen CX, et al. Combined venomics, antivenomics and venom gland transcriptome analysis of the monocled cobra (*Naja* *kaouthia*) from China. J Proteomics. 2017;159: 19-31.

Yalcin HT, Ozen MO, Gocmen B, Nalbantsoy A. Effect of Ottoman viper (*Montivipera* *xanthina* (Gray, 1849)) venom on various cancer cells and on microorganisms. Cytotechnology. 2014;66: 87-94.

Yang ZM, Yang YE, Chen Y, Cao J, Zhang C, Liu LL, et al. Transcriptome and proteome of the highly neurotoxic venom of *Gloydius intermedius*. Toxicon. 2015;107: 175-186.

Zainal Abidin S, Rajadurai P, Chowdhury M, Ahmad Rusmili M, Othman I, Naidu R. Proteomic characterization and comparison of malaysian *Tropidolaemus wagleri* and *Cryptelytrops purpureomaculatus* venom using shotgun-proteomics. Toxins. 2016;8: 299.

Zhang H, Yang Q, Sun M, Teng M, Niu L. Hydrogen peroxide produced by two amino acid oxidases mediates antibacterial actions. J Microbiol. 42: 336-339.

Zhao H, Gan TX, Liu XD, Jin Y, Lee WH, Shen JH, et al. Identification and characterization of novel reptile cathelicidins from elapid snakes. Peptides. 2008;29: 1685-1691.
